# Mental health and stress among ICU healthcare professionals in France according to intensity of the COVID-19 epidemic

**DOI:** 10.1186/s13613-021-00880-y

**Published:** 2021-06-04

**Authors:** Alexandra Laurent, Alicia Fournier, Florent Lheureux, Guillaume Louis, Saad Nseir, Gwenaelle Jacq, Cyril Goulenok, Grégoire Muller, Julio Badie, Bélaïd Bouhemad, Marjolaine Georges, Paul-Michel Mertes, Hamid Merdji, Vincent Castelain, Caroline Abdulmalak, Olivier Lesieur, Gaëtan Plantefeve, Jean-Claude Lacherade, Jean-Philippe Rigaud, Nicholas Sedillot, Damien Roux, Nicolas Terzi, Pascal Beuret, Antoine Monsel, Anne-Laure Poujol, Khaldoun Kuteifan, Thierry Vanderlinden, Anne Renault, Bérengère Vivet, Christophe Vinsonneau, Saber Davide Barbar, Gilles Capellier, Jean Dellamonica, Stephan Ehrmann, Thomas Rimmelé, Julien Bohé, Pierre Bouju, Sébastien Gibot, Bruno Lévy, Johanna Temime, Cyrille Pichot, David Schnell, Diane Friedman, Pierre Asfar, Eddy Lebas, Philippe Mateu, Kada Klouche, Juliette Audibert, Fiona Ecarnot, Nicolas Meunier-Beillard, Mélanie Loiseau, Irène François-Pursell, Christine Binquet, Jean-Pierre Quenot, PsyCOVID-ICU Trial Investigators and the CRICS TRIGGERSEP Group (Clinical Research in Intensive Care and Sepsis Trial Group for Global Evaluation and Research in Sepsis)

**Affiliations:** 1 Laboratoire de Psychologie: Dynamiques Relationnelles et Processus Identitaires (PsyDREPI), Université de Bourgogne Franche-Comté, Dijon, France; 2 Department of Anaesthesiology and Critical Care Medicine, Dijon University Medical Centre, Dijon, France; 3 Laboratoire de Psychologie, Université de Bourgogne Franche-Comté, Besançon, France; 4 Service de Réanimation Polyvalente et USC, Hôpital de Mercy, CHR Metz-Thionville, Thionville, France; 5 Critical Care Center, CHU Lille and Lille University, Lille, France; 6 Medical‐Surgical Intensive Care Unit, CH de Versailles, Le Chesnay, France; 7 Medical-Surgical Intensive Care Unit, Ramsay Générale de Santé, Hôpital Privé Jacques Cartier, Massy, France; 8 Service de Médecine Intensive-Réanimation, CHR d’Orléans, Orléans, France; 9 Service de Réanimation Polyvalente-USC, Hôpital Nord Franche-Comté, Trevenans, France; 10 Service de Pneumologie, CHU Dijon, Dijon, France; 11 Department of Anesthesia and Intensive Care, Hôpitaux Universitaires de Strasbourg, Nouvel Hôpital Civil, Strasbourg, France; 12 Faculté de Médecine, Université de Strasbourg (UNISTRA), Strasbourg, France; 13 Service de Médecine Intensive-Réanimation, Hôpitaux Universitaires de Strasbourg, Nouvel Hôpital Civil, Strasbourg, France; 14 Hôpitaux Universitaires de Strasbourg, Médecine Intensive – Réanimation, Hôpital de Hautepierre, Strasbourg, France; 15 Fédération de Médecine Translationnelle de Strasbourg, Faculté de Médecine, Université de Strasbourg, Strasbourg, France; 16 Service de Médecine Intensive-Réanimation, CH de Chalon sur Saône, Chalon sur Saône, France; 17 Intensive Care Unit, Groupement Hospitalier La Rochelle-Ré-Aunis, La Rochelle, France; 18 Service de Réanimation Polyvalente et USC, Argenteuil, France; 19 Service de Médecine Intensive-Réanimation, CH de La Roche-sur-Yon, Chalon sur Saône, France; 20 Service de Médecine Intensive-Réanimation, CH de Dieppe, Dieppe, France; 21 Espace de Réflexion Éthique de Normandie, Université de Caen, Caen, France; 22 Réanimation Polyvalente, CH de Bourg-en-Bresse, Bourg-en-Bresse, France; 23 Service de Médecine Intensive Réanimation, Assistance Publique - Hôpitaux de Paris, Hôpital Louis Mourier, Colombes, France; 24 Université de Paris, INSERM, UMR 1137 Infection, Antimicrobials, Modelling, Evolution, Paris, France; 25 Service de Réanimation Médicale, CHU de Grenoble, Grenoble, France; 26 Service de Réanimation-Soins Continus du CH de Roanne, Roanne, France; 27 Multidisciplinary Intensive Care Unit, Department of Anesthesiology and Critical Care, La Pitié-Salpêtrière Hospital, Assistance Publique-Hôpitaux de Paris, Sorbonne University, Paris, France; 28 Sorbonne University, INSERM, UMR-S 959, Immunology-Immunopathology-Immunotherapy (I3), Paris, France; 29 Biotherapy (CIC-BTi) and Inflammation-Immunopathology-Biotherapy Department (DHU i2B), Hôpital Pitié-Salpêtrière, AP-HP, Paris, France; 30 Équipe VCR, École de Psychologues Praticiens, Université Catholique de Paris, EA, 7403 Paris, France; 31 Laboratoire APEMAC, Université de Lorraine, EA 4360, Université́ de Lorraine, Metz, France; 32 Service de Réanimation Médicale, CH de Mulhouse, France; 33 Service de Médecine Intensive Réanimation, Groupe des Hôpitaux de L’Institut Catholique de Lille (GHICL), France, Université Catholique de Lille, Lille, France; 34 Service de Réanimation Médicale et Urgences Médicales, CHU de Brest, Brest, France; 35 Service de Réanimation Polyvalente, Groupe Hospitalier Intercommunal de La Haute-Saône, Site de Vesoul, Luxeuil-les-Bains, France; 36 Service de Médecine Intensive Réanimation-Unité de Sevrage Ventilatoire et Réhabilitation, CH de Bethune, Bethune, France; 37 Service des Réanimations, Faculté de Médecine de Montpellier-Nîmes, CHU de Nîmes, France and Université de Montpellier, Nîmes, France; 38 Réanimation Médicale, University Hospital Besançon, Besançon, France; 39 EA3920, University of Burgundy-Franche-Comté, Besançon, France; 40 Medical ICU University Hospital of Nice/UR2CA, Tours, France; 41 Service de Médecine Intensive-Réanimation, Tours, France; 42 CIC INSERM 1415, CRICS-TriggerSep Network, Tours, France; 43 INSERM, Centre d’étude des pathologies respiratoires, Université de Tours, U1100 Tours, France; 44 Anesthesiology and Intensive Care Medicine, Edouard Herriot Hospital, Lyon, France; 45 Service D’anesthésie - Réanimation-Médecine Intensive, CH Lyon-Sud, Hospices Civils de Lyon, Pierre Bénite, Tours, France; 46 Service Réanimation Polyvalente, Groupe Hospitalier Bretagne Sud, Lorient, France; 47 Service de Réanimation Médicale, Hôpital Central, Nancy, France; 48 Service de Réanimation Médicale, Centre Hospitalier Universitaire Nancy Brabois, Nancy-France-Institut du Cœur et des Vaisseaux. Groupe Choc, équipe 2, Inserm U1116. Faculté de Médecine, Nancy-Brabois, France; 49 Service de Réanimation Polyvalente, Lens, France; 50 Unité de Surveillance Continue, CH de Dôle, Dôle, France; 51 Service de Réanimation Polyvalente et USC, CH d’Angoulême, Angoulême, France; 52 Service de Médecine Intensive et Réanimation, Hôpital Raymond Poincaré, Garches, France; 53 Département de Médecine Intensive-Réanimation, CHU Angers, Angers, France; 54 Service de Réanimation-USC de Bretagne Atlantique, Vannes, France; 55 Service de Médecine Intensive-Réanimation-Unité de Recherche Clinique Ardennes Nord, CH de Charleville-Mézieres, Charleville-Mézieres, France; 56 Intensive Care Medicine Department, Lapeyronie Hospital, University Hospital of Montpellier-PhyMedExp, University of Montpellier, INSERM, CNRS, Montpellier, France; 57 Service de Réanimation Polyvalente, CH de Chartres, Hôpital Louis Pasteur, Le Coudray, France; 58 Department of Cardiology, University Hospital, Besançon, and EA3920, University of Burgundy-Franche-Comté, Besançon, France; 59 CIC 1432, Clinical Epidemiology, University of Burgundy, Dijon, France; 60 DRCI, USMR, Francois Mitterrand University Hospital, Dijon, France; 61 Service de Médecine Légale CHU Dijon, Cellule D’Urgence Médico-Psychologique de Bourgogne Franche-Comté, Dijon, France; 62 Inserm et CHU Dijon-Bourgogne, CIC1432, Module Epidémiologie Clinique, Dijon, France; 63 Service de Médecine Intensive-Réanimation, CHU Dijon-Bourgogne, France-Equipe Lipness, centre de recherche INSERM UMR1231 et LabEx LipSTIC, Université de Bourgogne-Franche Comté, Dijon, France; 64 INSERM, Module Épidémiologie Clinique, Université de Bourgogne Franche-Comté, CIC 1432, Dijon, France; 65 Espace de Réflexion Éthique Bourgogne Franche-Comté (EREBFC), Besançon, France; 66 Critical Care Department, University Hospital François Mitterrand, 14 rue Paul Gaffarel, 21079 Dijon, France

**Keywords:** Mental health, Stress factors, ICU, Healthcare professionals, COVID-19

## Abstract

**Background:**

We investigated the impact of the COVID-19 crisis on mental health of professionals working in the intensive care unit (ICU) according to the intensity of the epidemic in France.

**Methods:**

This cross-sectional survey was conducted in 77 French hospitals from April 22 to May 13 2020. All ICU frontline healthcare workers were eligible. The primary endpoint was the mental health, assessed using the 12-item General Health Questionnaire. Sources of stress during the crisis were assessed using the Perceived Stressors in Intensive Care Units (PS-ICU) scale. Epidemic intensity was defined as high or low for each region based on publicly available data from Santé Publique France. Effects were assessed using linear mixed models, moderation and mediation analyses.

**Results:**

In total, 2643 health professionals participated; 64.36% in high-intensity zones. Professionals in areas with greater epidemic intensity were at higher risk of mental health issues (*p* < 0.001), and higher levels of overall perceived stress (*p* < 0.001), compared to low-intensity zones. Factors associated with higher overall perceived stress were female sex (*B* = 0.13; 95% confidence interval [CI] = 0.08–0.17), having a relative at risk of COVID-19 (*B* = 0.14; 95%-CI = 0.09–0.18) and working in high-intensity zones (*B* = 0.11; 95%-CI = 0.02–0.20). Perceived stress mediated the impact of the crisis context on mental health (*B* = 0.23, 95%-CI = 0.05, 0.41) and the impact of stress on mental health was moderated by positive thinking, *b* = − 0.32, 95% CI = − 0.54, − 0.11.

**Conclusion:**

COVID-19 negatively impacted the mental health of ICU professionals. Professionals working in zones where the epidemic was of high intensity were significantly more affected, with higher levels of perceived stress. This study is supported by a grant from the French Ministry of Health (PHRC-COVID 2020).

**Supplementary Information:**

The online version contains supplementary material available at 10.1186/s13613-021-00880-y.

## Introduction

The rapid spread of the SARS-CoV-2 epidemic in France in March 2020 led to the implementation of the national emergency preparedness plan in French hospitals. The plan involves wide-scale reorganization of healthcare institutions to respond to the massive influx of patients. Prior to the epidemic, there were 5432 available intensive care unit (ICU) beds, whereas during the peak of the epidemic in France, more than 80,000 patients were hospitalized and 13,677 required intensive care [1]. This massive and increasing influx of contaminated patients put hospitals, and particularly ICUs, under extreme tension. Even before the epidemic, the working conditions in intensive care were already identified as stressful for professionals, in particular due to the constant technological progress, the end-of-life challenges, issues relating to organ retrieval, high workload and night shifts [2, 3]. The COVID crisis added an additional degree uncertainty and insecurity, and was unprecedented for healthcare workers [4, 5]. It was also compounded by the risk of being contaminated or contaminating others, as well as by a lack of personal protective equipment for personnel, and a shortage of healthcare workers trained in intensive care. The lack of specific treatment for the infection, and the wide variability in the course of the disease contributed to generating contradictory information, forcing healthcare professionals to readjust management strategies constantly. Finally, the question of bed availability and the risk of patient triage raised numerous ethical and moral dilemmas within the caregiving teams, where the principles of collegiality and family presence were undermined [6].

The psychological impact of this health crisis on healthcare workers is a major concern. Studies conducted on the COVID-19 epidemic in China [7, 8], and in previous epidemics, e.g., H1N1 (2009) and SARS-CoV-1 (2003), reported that frontline professionals may show symptoms of anxiety, depression, sleep disorders, post-traumatic stress disorder and burnout [9–13], and symptoms may persist long after the crisis [11, 12]. A cross-sectional survey during the current pandemic among intensivists in Europe reported prevalence of anxiety, depression or severe burnout of 46.5%, 30.2%, and 51%, respectively, with significant variation across regions [14].

We report here the results of a French multicenter, cross-sectional survey (Psy-COVID-ICU [Psychological support for health care professions in intensive care units in the COVID-19 pandemic context]) that aimed to identify the impact of the crisis on the mental health of ICU professionals according to the intensity of the epidemic in France. We also examined whether job stress, coping strategies and socio-demographic characteristics of ICU professionals were associated with mental health.

## Methods

### Study design and oversight

The PsyCOVID-ICU study was conducted in 77 hospitals across France from 22 April 2020, to 13 May 2020. The complete list of participating sites is provided in Additional file 1: Appendix Table S1.

This study received approval for all participating centers from the Ethics Committee of the French Intensive Care Society (No 20–33). The study was overseen by a trial management committee. The first author drafted the manuscript, which was reviewed by the trial management committee. The authors attest that the study was performed in accordance with the protocol and vouch for the accuracy and completeness of the reported analyses. There was no commercial support for the trial. This study was supported by a grant from the French Ministry of Health (PHRC-COVID 2020).

## Study procedures

### Participants

The population study comprised ICU frontline healthcare workers directly involved in the diagnosis, treatment, and care of patients with COVID-19 (i.e., physicians, residents, nurses, nurses’ aides, medical students and nursing managers) who consented to participate.

An online questionnaire was implemented using the Limesurvey platform. Health care professionals were informed orally and via posters about the study objectives and procedures. The posters gave the link to access the questionnaire. Healthcare professionals were required to read and accept the terms of the study briefing note before starting to respond. Responses to the questionnaires were anonymous and confidential. The Chief of each participating ICU completed a questionnaire concerning the characteristics and volume of activity of their center. Each individual participant completed demographic data (Additional file 1: Appendix, Box S1).

### Study outcomes

The primary endpoint was mental health, as assessed by the validated French version of the 12-item General Health Questionnaire (GHQ-12) [15]. The GHQ-12 is a self-report measure of the severity of psychological morbidity in non-psychiatric settings [16, 17]. It measures change in mental state following upsetting events, by assessing symptoms related to psychological distress and general functioning [18]. We used the standard scoring method (0–0–1–1), which gives a possible score ranging from 0 to 12. We used the total score, as recommended by Hystad and Johnsen [19], a higher score indicates a greater degree of psychological distress. A threshold of 3 or more has been used to identify the presence of distress in other studies [20–22]. To measure sources of stress in ICU during the COVID-19 crisis, we used 13 items specific to the epidemic context, taken from a scale developed by Khalid et al. during the 2015 MERS-CoV outbreak in Saudi Arabia [23] and 27 items from the Perceived Stressors in Intensive Care Units (PS-ICU) scale [24, 25]. Details of how these 27 items were selected are given in Additional file 1: Appendix, Box S2 and Table S2. From these items, we performed factor analysis with Oblimin rotation. Principal axis factoring revealed a factorial structure in six dimensions, namely one dimension specific to COVID-19 stressors (D1; e.g., “You developed respiratory symptoms and feared that you had COVID-19”), Patient- and family-related emotional load dimension (D2; e.g., “Death of a patient with whom I had developed special ties”), Complex/risky situations and skill-related issues dimension (D3; e.g., “Treating complex or serious pathologies”), Workload and human-resource management issues dimension (D4; e.g., “Continuous and heavy workload”), Difficulties related to the team-working dimension (D5; e.g., “Conflicts with members of the healthcare team”), and Care provided in sub-optimal or conflictual conditions dimension (D6; e.g., “Shortage of beds in the unit”) (Additional file 1: Tables S3 and S4). Items were rated on a 5-point Likert scale ranging from 0 (“I didn’t experience this situation”) to 4 (“I experienced this situation, and I was very much stressed”). An overall perceived stress score was calculated from the 40 items ranging from 0 to 160. Next, we assessed coping strategies using the Brief-COPE questionnaire [26]. Four types of coping were assessed (social support seeking, problem solving, avoidance and positive thinking) that are likely to act as a buffer against stressful events [27, 28]. Higher scores reflect a greater tendency to implement the corresponding coping strategy.

### Definition of epidemic intensity

The intensity of the epidemic for each region was defined according to the ratio of the maximum number of patients in the ICU during the 1st wave of the pandemic in each administrative area (department) (public data provided by the French public health agency, Santé Publique France—https://geodes.santepubliquefrance.fr/#c=home) to the maximum number of ICU beds available in the same area before the crisis (Statistique Annuelle des Etablissements (SAE) 2018 provided by the Direction de la Recherche des études, de l'évaluation et des statistiques (DREES)). High-intensity zones were defined as those with a ratio > 1. Zones with a ratio < 1 were classed as low-intensity.

### Statistical analysis

Quantitative variables are described as mean ± SD and categorical variables as number (percentage). We compared GHQ-12 and perceived stress scores between areas with high and low epidemic intensity, using the z test or Welch’s F and Tukey statistics, as appropriate. To identify factors associated with perceived stress intensity, linear mixed effects modeling using the restricted maximum likelihood method was used via the lmer function in the R package lme4 [29, 30]. We estimated the effect of the epidemic intensity (high- vs. low-intensity zone) on the perceived stress of all professionals (as assessed by the composite scale comprising Khalid’s 13 items and 27 PS-ICU items), controlling for gender, having a family member at risk of developing COVID-19 (due to the presence of a chronic comorbidity or advanced age putting them at risk of a severe form of COVID-19), being a professional who usually works in ICU (versus professionals who usually work in other units, but were requisitioned or volunteered to work in the ICU during the crisis), and number of years’ experience (i.e., < 5 years, 5 to 10 years, > 10 years) in the occupation as fixed factors. Because the interclass correlation coefficients indicated that the variance in perceived stress could be attributed to differences between hospitals and occupational status (respectively, 7.9% and 7.6%), and because the likelihood ratio test statistics (LR) for each variable were significant (*p* < 0.05), we included hospitals and occupational status as random effects to take account of heterogeneity across clusters of participants. We selected the final model with the best fit and maintaining model parsimony using the Akaike Information Criterion (AIC) [31]. Finally, to explore the relation between stress intensity and mental health across zones with different levels of epidemic intensity, we performed causal mediation analysis of multilevel data via the lmer function in the lme4 package [29] and the mediate function in the mediation package [32]. The impact of stress on overall health depends on the use of specific coping strategies [27]. Thus, we analyzed whether the intensity of the epidemic (high- vs. low-intensity zone) affected the mental health of ICU professionals, as measured by the GHQ-12 score, and hypothesized that the total stress measured by the perceived stress scale (Khalid and 27-item PS-ICU composite scale) functioned as the causal mechanism, with the effects on mental health being mediated by the use of the coping strategies measured by the Brief-COPE. We also introduced gender, having a family member at risk of developing COVID-19, being a professional who usually works in ICU, length of experience in the occupation, and occupational status as covariates and fixed variables. Hospital affiliation was introduced as a random variable to control for heterogeneity. We proceeded in the same manner for each dimension of the stress scale.

All data analyses were performed using R (version 4.0.3) and its interface R studio server (version 1.4.1103), and SPSS (version 26) for Macintosh. The significance threshold was set at *p* < 0.05.

## Results

### Demographic characteristics

**Fig. 1:**
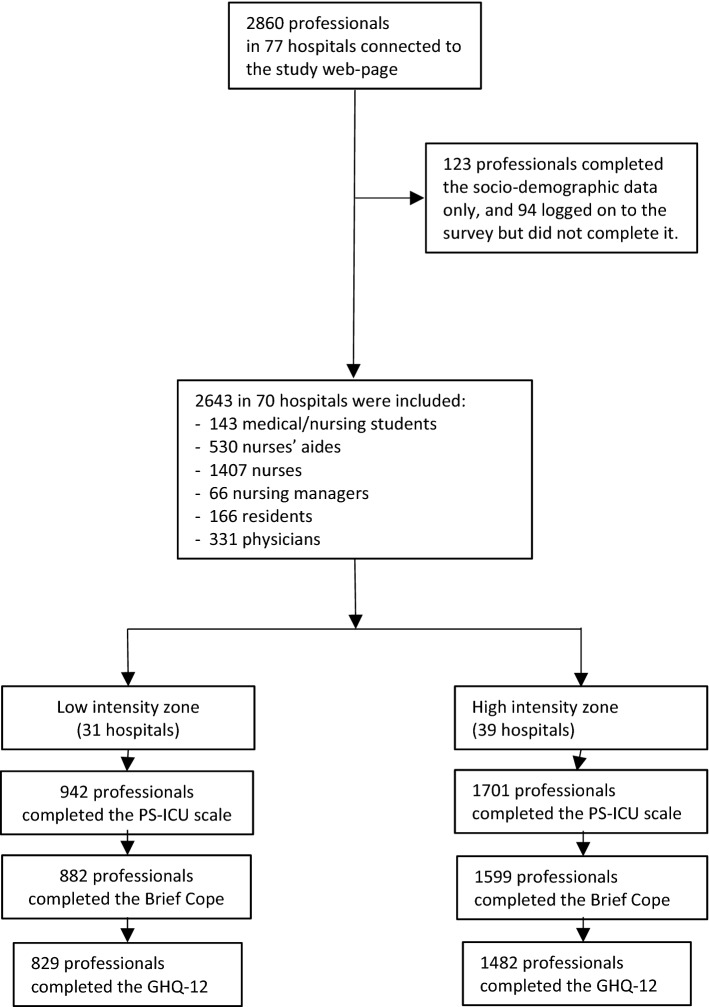
Study assignment and follow-up

A total of 2,643 healthcare professionals were included in this study, 1920 (72.6%) were women, more than half the population were nurses (1407, 53.2%) (Fig. 1—study flowchart). In total, 942 (35.64%) participants in low-intensity zones and 1701 (64.36%) in high-intensity zones (Table 1).

**Table 1:** Sociodemographic data of the overall population and according to epidemic intensity zone

	Epidemic intensity	
	Low (*n*, %)	High (*n*, %)
Overall	942 (100)	1701 (100)
Number of hospitals	31	39
Sex		
Women	715 (75.9)	1205 (70.84)
Men	227 (24.1)	496 (29.16)
Age, years		
20–34	458 (48.62)	914 (53.73)
35–49	396 (42.04)	622 (36.57)
50–65	88 (9.34)	160 (9.41)
> 65	0 (0)	5 (0.29)
Occupational status		
Healthcare students	40 (4.25)	103 (6.06)
Nurses’ aides	222 (23.57)	308 (18.11)
Nurses	515 (54.67)	892 (52.44)
Nursing managers	27 (2.87)	39 (2.29)
Residents	52 (5.52)	114 (6.7)
Physicians	86 (9.13)	245 (14.4)
Marital status		
Single/divorced/separated/widowed	267 (28.34)	540 (31.75)
Married/living maritally	668 (70.91)	1142 (67.14)
Missing data	7 (0.74)	19 (1.12)
Increase in working time compared to usual		
Yes	382 (40.55)	935 (54.97)
No	512 (54.35)	692 (40.68)
Missing data	48 (5.1)	74 (4.35)
Duration of work experience		
< 5 years	243 (25.8)	545 (32.04)
5 to 10 years	276 (29.3)	458 (26.93)
> 10 years	408 (43.31)	667 (39.21)
Missing data	15 (1.59)	31 (1.82)
Working hours		
Part-time	97 (10.3)	204 (11.99)
Full-time	827 (87.79)	1470 (86.42)
Missing data	18 (1.91)	27 (1.59)
Pre-COVID position in ICU		
Yes	737 (78.24)	1199 (70.49)
No	205 (21.76)	502 (29.51)
COVID risk for a relative		
Yes	639 (67.83)	1199 (70.49)
No	279 (29.62)	459 (26.98)
Missing data	24 (2.55)	43 (2.53)

*ICU* intensive care unit

### Impact of epidemic intensity on mental health

Compared with professionals working in low-intensity zones, professionals in high-intensity zones had a significantly higher GHQ-12 score (*p* ≤ 0.001). Professionals in all zones had an average GHQ-12 score > 3, indicating the presence of distress, regardless of epidemic intensity (Table 2).

**Table 2:** Average scores on the study questionnaires among all professionals

	Epidemic intensity		Welch’s *F*	*p*
	Low	High		
Mental health (/12)	3.86 (2.86)	4.33 (3.09)	13.43	** < 0.001**
Overall perceived stress (/4)	1.48 (0.52)	1.57 (0.54)	15.54	** < 0.001**
Dimension 1 (/4)	2.1 (0.75)	2.14 (0.76)	1.67	0.197
Dimension 2 (/4)	1.36 (0.81)	1.51 (0.82)	21.34	** < 0.001**
Dimension 3 (/4)	1.84 (0.76)	1.86 (0.73)	0.45	0.502
Dimension 4 (/4)	1.46 (0.73)	1.57 (0.77)	14.34	** < 0.001**
Dimension 5 (/4)	0.95 (0.87)	0.99 (0.91)	1.19	0.275
Dimension 6 (/4)	0.68 (0.73)	0.85 (0.75)	34.81	** < 0.001**
Coping strategies				
Social support (/4)	2.58 (0.82)	2.61 (0.84)	1.03	0.311
Problem solving (/4)	2.63 (0.92)	2.7 (0.92)	2.91	0.088
Avoidance (/4)	2.53 (0.97)	2.66 (0.96)	11.58	**0.001**
Positive thinking (/4)	2.86 (0.9)	2.82 (0.91)	1.2	0.273

Mean and standard deviation are reported. Dimension 1: COVID-19 specific stressors; Dimension 2: patient- and family-related emotional load; Dimension 3: complex/risky situations and skill-related issues; Dimension 4: workload and human resources management issues; Dimension 5: difficulties related to the team-working; Dimension 6: care provided in sub-optimal or conflictual conditions; PSI-CU: perceived stress scale in intensive care unit; GHQ-12: 12-item General Health Questionnaire

### Impact of epidemic intensity on perceived stress

Compared to professionals working in low-intensity zones, professionals in high-intensity zones had higher levels of overall perceived stress, as assessed by the stress scale (*p* ≤ 0.001). In particular, they exhibited more stress related to workload and human resources management issues (Dimension 4) (*p* ≤ 0.001), care provided in sub-optimal or conflictual conditions (Dimension 6) (*p* ≤ 0.001) and more stress related to the emotional burden of the patient and family (Dimension 2) (*p* ≤ 0.001) (Table 2). Average scores for the dimension “COVID-19 specific stressors” (Dimension 1) were significantly higher than the average scores for the other dimensions in all intensity zones (*p* ≤ 0.001 for all), despite the lack of difference in dimension “COVID-19 specific stressors” between high- and low-intensity zones (*p* = 0.197) (Additional file 1: Appendix Figs. S1 and S2).

### Factors associated with job stress during the crisis

Linear mixed model analysis (Table 3) showed that, after controlling for hospital and occupational status, being a woman (*B* = 0.13; 95% confidence interval [CI], 0.08 to 0.17), having a relative at risk of developing COVID-19 (*B* = 0.14; 95% CI, 0.09 to 0.18) and working in a high-intensity zone were associated with higher overall perceived stress (*B* = 0.11; 95% CI, 0.02 to 0.20). Being an ICU professional (compared to staff from other departments requisitioned to work in the ICU during the crisis) had a protective effect (*B* = − 0.12; 95% CI, − 0.17 to − 0.07). Analysis of residuals associated with occupational categories indicated that physicians had the lowest level of perceived stress, whereas nurses had the highest level of perceived stress [physicians (mean score (M) = 1.42) < nursing managers (M = 1.43) < medical students (M = 1.45) < residents (M = 1.48) < nurses’ aides (M = 1.56) < nurses (M = 1.58)].

**Table 3:** Results from the linear mixed effects models for total stress scores

Overall perceived stress (AIC = 3781)	*B*	St err	*t* value	*p*	CI	
Female	0.13	0.02	5.23	**<** **0.001**	0.08	0.17
ICU professional	− 0.12	0.02	− 5.01	**<** **0.001**	− 0.17	− 0.07
Relatives with risk	0.14	0.02	6.13	**<** **0.001**	0.09	0.18
Experience 5 to 10 years	0.02	0.03	0.84	0.407	− 0.03	0.08
Experience >10 years	− 0.04	0.03	− 1.67	0.101	− 0.09	0.01
Epidemic intensity zone	0.11	0.05	2.37	**0.023**	0.02	0.20

*St err* standard error,* CI* confidence interval

### Relation between epidemic intensity, coping strategies and mental health

When we compared the use of coping strategies across zones of epidemic intensity, the analyses showed that avoidance was the only coping strategy that was used more frequently by healthcare professionals working in zones with high epidemic intensity, as compared to low-intensity zones (*p* = 0.001) (Table 2).

We also analyzed the correlations between GHQ-12, and scores on the perceived stress scale and coping strategies. There was a positive correlation between GHQ-12 and the scores from the perceived stress scale, with each type of stressor contributing significantly to explaining the severity of psychological distress (0.20 ≤ *r* ≤ 0.45, *p* < 0.001), and the total score had the strongest correlation (0.52, *p* < 0.001). Furthermore, there was a positive correlation between GHQ-12, and each of the following coping strategies: social support, problem solving, and avoidance (0.19 ≤ *r* ≤ 0.36, *p* < 0.001). There was a negative correlation between the positive reframing coping strategy and psychological distress (*r* = − 0.16, *p* < 0.001) (Additional file 1: Appendix, Table S9).

**Fig. 2:**
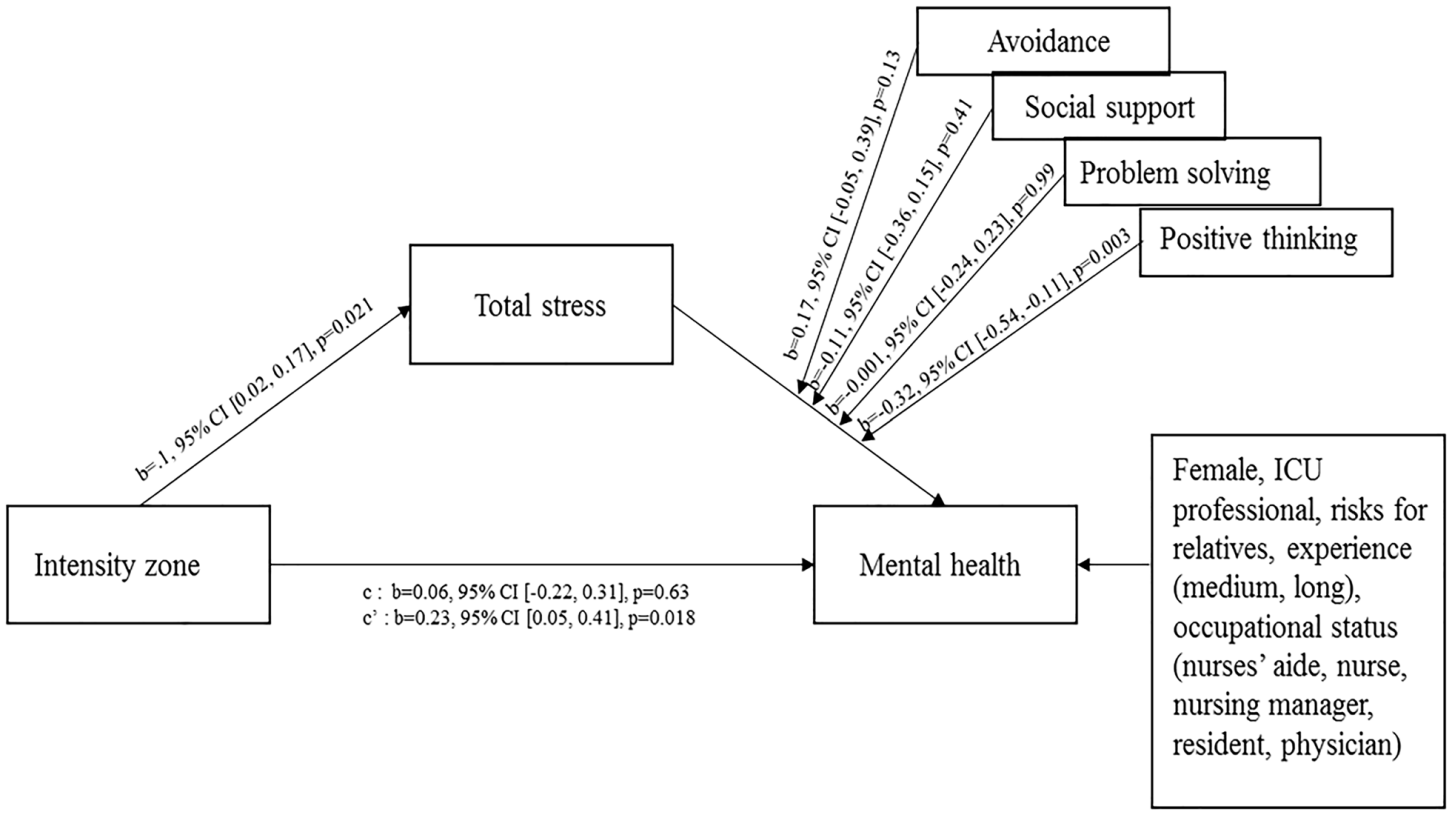
Mediation and moderation analysis of the relationship between the epidemic intensity zone and mental health through total stress scores. *ICU professional: *b* = 0.88, [0.63, 1.12], *p* < 0.001

Mediation and moderation analyses including hospital as a random variable revealed a significant indirect effect of the epidemic intensity on psychological distress, via the overall perceived stress score, (*b* = 0.23, 95% CI [0.05, 0.41]). Positive thinking coping strategies significantly moderated the association of stress with mental health issues, *b* = − 0.32, 95% CI [− 0.54, − 0.11], whereby the more professionals used this type of coping, the smaller the association between job stress and their psychological distress. None of the other coping strategies was found to have a moderating effect. In addition, being an ICU professional (compared to staff from other departments requisitioned to work in the ICU during the crisis) was associated with an increase in the effect of epidemic intensity on psychological distress, *b* = 0.88, [0.63, 1.12] (Fig. 2). Among the six specific stress dimensions measured by the stress scale, the effect of the epidemic intensity on mental health was mainly driven by dimension 2 (patient- and family-related emotional load, *b* = 0.13, 95% CI [0.03, 0.22]) and dimension 6 (care provided in sub-optimal or conflictual conditions, *B* = 0.09, 95% CI [0.02, 0.18]) (Additional file 1: Appendix Figs. S3 to S8).

## Discussion

This multicenter study involving 2,643 ICU professionals during the COVID-19 epidemic in France showed that the intensity of the epidemic had an impact on the mental health of healthcare professionals. Professionals working in heavily impacted hospitals, in zones where the epidemic was of high intensity showed greater psychological distress linked to the crisis than healthcare professionals located in low-intensity zones. This psychological distress can be explained by the intensity of the job stress felt by healthcare professionals; the greater the stress, the greater the psychological distress. The impact of the crisis on the mental health of healthcare professionals was mainly explained by work situations related to the emotional intensity of the relationship with the patient and the family (Dimension 2: death, announcing a diagnosis, patient/family distress), and the feeling of inadequate and sub-optimal care (Dimension 6: lack of beds, conflicts in patient care and in the information given to the family).

In addition, regardless of the intensity of the epidemic, the mental health of the professionals working in ICUs was affected, with marked psychological distress. Our results showed the generalized state of tension among ICU professionals in France. Whatever the geographical area, situations related to COVID-19 (fear of transmission, fear of being contaminated or infecting others, lack of information, protection, treatment, etc.) are the sources of stress most intensely felt by healthcare professionals.

Our study also highlights several factors during the epidemic crisis that put healthcare professionals at risk of developing psychological distress and high levels of stress. The socio-professional categories in closest contact with patients, namely nurses, residents, and nurses’ aides, showed the highest levels of stress. Professionals who were not permanent members of the ICU staff (i.e., temporary support staff), women, and those with a relative at risk of developing complications during COVID-19 were also at higher risk. These results are consistent with the study by Lai et al. [8] showing that being a woman and a nurse during the COVID-19 epidemic in China was associated with severe symptoms of depression, anxiety, and distress, and the study by Azoulay et al. showing that female gender was significantly associated with both anxiety and depression [14]. In addition, nurses have been identified as one of the professions with the highest level of stress in their daily work, outside the pandemic context [33–36]. However, this is likely explained by the fact that nurses are predominantly female, and tend to have high levels of perceived stress [37] given the demands of their profession, notably conflicts between the home life and work life [38]. A study by Khalid et al. [23] also underlined the importance of the stress caused by the fear of contaminating relatives. Thus, the COVID-19 crisis affects both the private and professional spheres, leading to a constant perception of risk.

To protect themselves from the crisis, healthcare professionals working in the most impacted areas most frequently used avoidance strategies, ranging from cognitive avoidance and resignation, to seeking satisfaction in order to stop thinking about the crisis. However, the coping strategy that was most effective in reducing the stress felt during the crisis was "positive thinking", as shown in our moderate mediation analysis. Positive thinking is a cognitive process that helps individuals to deal with stress more effectively, by enabling the person not only to look at the negative aspects of an event, but also the positive aspects, and to focus on a positive interpretation [39, 40]. Many aspects of the COVID-19 epidemic crisis are beyond the control of professionals, with a high level of uncertainty, so this strategy seems particularly well suited. Indeed, while the caregiver cannot change the situation, they can change their interpretation/representation by focusing on positive cognition that promotes positive mental states [41–43]. Targeted strategies focusing on positive thinking, notably in terms of positive leadership approaches by department chiefs and nursing managers, could help to mitigate the stress perceived by frontline healthcare workers in the epidemic context [44]. Regarding the coping strategy that consists in seeking social support, although it was not found here to have a moderating effect in the relation between epidemic intensity and mental health, it was nonetheless correlated with psychological distress. This is surprising, since research into social support shows that it generally tends to help preserve the mental health of those facing difficult situations [45]. However, the unique nature of the current pandemic context, with stress running high in both the personal and professional spheres, may have resulted in some negative emotional contagion in the close environment of these healthcare professionals [46]. Indeed, this finding has previously been reported in other studies [47, 48], but should be interpreted with caution here notably due to the cross-sectional nature of our study.

Our study has some limitations. Firstly, since only ICU professionals participated in this study, our results may not be generalizable to other contexts. Second, a total of 2860 healthcare professionals opened the link to the study page during the study period, of whom 94 did not go on to complete the questionnaire, and 123 completed only the socio-demographic data. These participants chose to connect to the study online after receiving the study information disseminated by the Department Chiefs in 77 hospitals in France. However, the actual number of potentially eligible ICU professionals (i.e., the denominator) is unknown. Participants were not contacted individually by name with an email or letter that could be followed up by a reminder. Third, the study period was short, but corresponded to the peak of the first epidemic wave in France. Fourth, the stress scale used to evaluate stress in the ICU was developed recently, limiting the possibility for comparison with other studies. However, it enabled us to use an appropriate tool developed specifically for the ICU context, which best takes into account the unique features of the ICU environment. Fifth, only healthcare professionals in close contact with COVID patients were recruited for this study, and therefore, results may not be generalizable to other professions. Finally, we did not measure the baseline workload in the different zones, precluding any conclusion about whether the differences observed between high-intensity and low-intensity zones are due to unequal baseline workload. Similarly, different severity of the COVID patients may also have contributed to variations in baseline workload. However, the results obtained with the PS-ICU scale show that Factor 4 (workload and human resources management) did not have a significant effect, therefore suggesting that the baseline workload is not a major contributor to the differences observed.

In conclusion, the COVID-19 epidemic had a strong negative impact on the mental health of professionals working in the ICU. Healthcare professionals working in zones where the epidemic was of high intensity were significantly more affected, with higher levels of perceived stress. Healthcare professionals in closest contact with the patients had the highest levels of perceived stress, driven mainly by the emotional and ethical burden, and this can be alleviated by the use of coping strategies, particularly positive thinking.

## Supplementary Information

**Additional file 1.** Additional figures and tables.

## Data Availability

Data are available from the corresponding author on reasonable request.
