# Supplementary material for: Mental health and stress among ICU healthcare professionals in France according to intensity of the COVID-19 epidemic
Source: Ann Intensive Care. 2021 Jun 4;11:90. doi: 10.1186/s13613-021-00880-y (PMC8177250; doi:10.1186/s13613-021-00880-y)
Supplement: Supplementary file 1 — Additional file 1. Additional figures and tables. [file 13613_2021_880_MOESM1_ESM.docx]

**Mental health and stress among ICU caregivers in France according to intensity of the COVID-19 epidemic**

**A Laurent et al**

**Additional file 1: Appendix**

**Table Sof Contents**

[**Table S1. Number of participating centers by region** 3](#_Toc67058913)

[**Table S3. Principal axis analysis with Oblimin rotation with items from the Perceived stress in intensive care units scale (PS-ICU) [1,2] and items specific to COVID-19 from Khalid’s scale [3]** 11](#_Toc67058914)

[**Table S4. Items from the Perceived stress in intensive care units (PS-ICU) scale [1,2] and items specific to COVID-19 from Khalid’s scale [3]** 13](#_Toc67058915)

[**Table S5. Average scores obtained on the different scales according to epidemic intensity among students, nursing care assistants and nurses** 17](#_Toc67058916)

[**Table S6. Average scores obtained on the different scales according to epidemic intensity among Nursing managers, residents and physicians** 18](#_Toc67058917)

[**Table S7. Number of participants with a mental *health* disorder (GHQ-12 ≥ 3) according to occupation*al category* and epidemic intensity** 19](#_Toc67058918)

[**Table S8. Average scores on the different scales among professionals with a mental health disorder (GHQ-12 ≥ 3)** 20](#_Toc67058919)

[**Table S9. Correlations between questionnaire scores.** 21](#_Toc67058920)

[**Additional file 1: Figure S1.** 22](#_Toc67058921)

[**Additional file 1: Figure S2.** 23](#_Toc67058922)

[**Additional file 1: Figure S3.** 24](#_Toc67058923)

[**Additional file 1: Figure S4.** 25](#_Toc67058924)

[**Additional file 1: Figure S5.** 26](#_Toc67058925)

[**Additional file 1: Figure S6.** 27](#_Toc67058926)

[**Additional file 1: Figure S7.** 28](#_Toc67058927)

[**Additional file 1: Figure S8.** 29](#_Toc67058928)

[**Additional file 1: Appendix References** 30](#_Toc67058929)

# **Table S1. Number of participating centers by region**

| **Region** | **Number of centers** |
| --- | --- |
| Auvergne-Rhône-Alpes (Centre-East) | 11 |
| Bourgogne-Franche-Comté (North-East) | 8 |
| Bretagne (West) | 3 |
| Centre-val de Loire (Centre-West) | 6 |
| Grand-Est (North-East) | 11 |
| Haut de France (North) | 8 |
| Ile de France (Paris area) | 17 |
| Normandie (North-West) | 1 |
| Nouvelle Aquitaine (South-West) | 5 |
| Occitanie (South) | 2 |
| Provence-Alpes-Côte d'Azur (South-East) | 5 |

**Box 1. Questionnaire concerning the socio-demographic data**

|  |
| --- |

Questionnaire Professionnel

- Date of completion* : |__|__|/|__|__|/2020

- Name *(First 2 letters)* * : |__|__|

- First name *(first letter)* * : |__|

- Email address*: __________________________@______________

- Hospital name *(choose from drop-down menu)* **:* _______________________________________

- Sex *:  Male  Female

- Age *:  20-34 years  35-49 years  50-65 years  > 65 years

- Marital status :

*(Only 1 response possible)*

- Single
- Living maritally
- Married
- Separated
- Divorced
- Widowed

- Number of children : |__|__| of whom dependents: |__|__| [0-10]

- Living situation*:

- Living alone
- Living with family members *(spouse, children, ..)*
- Other: Please specify ________________________

- Place of residence at the time of completion of this survey :

- Home
- Accommodation close to the hospital

- Is anyone in your close entourage at risk of negative outcomes if they were to be infected by COVID-19 (e.g. hypertension, respiratory diseases) ?

  Yes  No

- Profession : *

- Qualified/full-time/contractual employee  Resident  Student
  - - Students, please specify: *: _________________________________________
    - Residents, please specify*: __________________________________________
    - Qualified/full-time/contractual employees, please specify*:

 Physician  Pharmacist  Social worker

 Nurse anesthetist  Nurse  Surgical nurse

 Nursing manager  Nurses’ aide  Orderly

 Physiotherapist  Other, please specify: ____________________________

- Years of experience in your current profession :

 < 5 years  5 to 10 years  > 10 years

- Working hours (currently) :

 Full time  Part-time  Specify : |__|__|__| % [20-90]

- Have your working hours increased because of the COVID-19 epidemic:  Yes  No

 If yes: - Number of extra hours per week: ______

- How are you rewarded for these extra hours:  Paid overtime

 Get time off in lieu

 No reward

- Number of night duties in the 7 days prior to completing the survey: |__|__| [0-4]

- Did you have postpone planned time off because of the crisis:  Yes  No

- Were you working in the ICU before the onset of the COVID-19 epidemic *:  Yes  No

 If yes: for how many months/years: |__|__| months OR |__|__| years

 If not, what department do you usually work in : _ _ _ _ _ _ _ _ _ _ _ _ _ _ _ _ _ _ _ _

- Are there any ongoing conflicts within your work team at the time of completion of this survey:  Yes  No

 If yes, please specify : _________________________________________________________________________

_______________________________________________________________________________________

Box 2.

Regarding the selection of the 6 dimensions of the PS-ICU scale retained in this study, we decided to use only part of the 50 items that compose the original scale, due to the addition of the 13 items from Khalid’s scale. To reduce the PS-ICU score, we first decided to conserve the 36 items that were considered to be the most important and the most sTable Sin previous factorial analyses. We then performed factorial analysis in 6 factors, using principal axis factoring and direct oblimin rotation. The aim was to conserve the four items with the highest factor loading, to obtain a 24-item scale. The reduction to 24 items led to the deletion of 4 items that were considered important (items 7, 44, 25 and 58), while one unimportant item was retained (item 8). Furthermore, factor analysis of the 24-item version revealed the existence of one factor containing unsTable Sitems via factor analysis for reduction (factor 6, items 8, 11, 12 and 32). We therefore decided to re-introduce the 4 important items, delete item 8, and force the factor analysis with 5 factors, again using the principal axis factoring with direct oblimin rotation. The results produced a 27-item scale whose items were sTable Sthroughout the reduction steps, and with homogeneous factors. After collecting data in the PsyCovid study, we performed factor analysis with principal axis factoring to verify the structure of the scale and found a 5-dimension structure that is sTable Sin comparison with previous results (cf Table S3 in the Additional file 1: Material).

The internal consistency parameters were satisfactory for all dimensions of the 27-item PS-ICU (Cronbach’s alpha >0.60 for all).

**Table S2.** **Principal axis analysis with Oblimin rotation with items from the Perceived Stress in Intensive Care Units scale (PS-ICU) to select 27 items**

| Items of PS-ICU | |  | **First step** | | | | | |  | **Second step** | | | | | |  | **Last step** | | | | |
| --- | --- | --- | --- | --- | --- | --- | --- | --- | --- | --- | --- | --- | --- | --- | --- | --- | --- | --- | --- | --- | --- |
|  |  |  | **F1** | **F2** | **F3** | **F4** | **F5** | **F6** |  | **F1** | **F2** | **F3** | **F4** | **F5** | **F6** |  | **F1 (𝛼=.787)** | **F2 (𝛼=.776)** | **F3 (𝛼=.792)** | **F4 (𝛼=.766)** | **F5 (𝛼=.706)** |
| 30 | Powerlessness or incompetence in supporting families |  | .36 |  |  |  |  |  |  |  |  |  |  |  |  |  |  |  |  |  |  |
| 7 | Families’ distress or emotions |  | .4 |  |  |  |  |  |  |  |  |  |  |  |  |  |  | .4 |  |  |  |
| 44 | Patient suffering physically or psychologically |  | .41 |  |  |  |  |  |  |  |  |  |  |  |  |  |  | .42 |  |  |  |
| 1 | Socially isolated end-of-life patient or one with no immediate family |  | .48 |  |  |  |  |  |  |  |  |  |  |  |  |  |  |  |  |  |  |
| 49 | Decision to stop or reduce treatment |  | .51 |  |  |  |  |  |  |  |  |  |  |  |  |  |  |  |  |  |  |
| 29 | Patient who makes me think of someone close to me or of myself |  | .55 |  |  |  |  |  |  |  |  |  |  | .47 |  |  |  | .53 |  |  |  |
| 34 | Death of a patient with whom I had developed special ties |  | .59 |  |  |  |  |  |  |  |  |  |  | .55 |  |  |  | .59 |  |  |  |
| 28 | Series of patient deaths in the unit over a short period |  | .61 |  |  |  |  |  |  |  |  |  |  | .62 |  |  |  | .63 |  |  |  |
| 45 | Having to announce a bad diagnosis to the patient or his/her family or be present when such a diagnosis is announced |  | .63 |  |  |  |  |  |  |  |  |  |  | .56 |  |  |  | .63 |  |  |  |
| 25 | Patient who deteriorates in an unexpected or unexplained manner |  |  | .42 |  |  |  |  |  |  |  |  |  |  |  |  | .38 |  |  |  |  |
| 37 | Having to perform tasks for which I have neither knowledge nor skills |  |  | .51 |  |  |  |  |  | .52 |  |  |  |  |  |  | .54 |  |  |  |  |
| 16 | Risk of error, fear of doing a poor job |  |  | .62 |  |  |  |  |  | .62 |  |  |  |  |  |  | .66 |  |  |  |  |
| 40 | Treating complex or serious pathologies |  |  | .67 |  |  |  |  |  | .69 |  |  |  |  |  |  | .64 |  |  |  |  |
| 18 | Having to execute care tasks quickly in emergency cases |  |  | .75 |  |  |  |  |  | .72 |  |  |  |  |  |  | .72 |  |  |  |  |
| 41 | Caring for a patient who should not be treated by the ICU |  |  |  | .3 |  |  |  |  |  |  |  |  |  |  |  |  |  |  |  |  |
| 26 | Family whose beliefs or lifestyle are contradictory with my values or the functioning of the unit |  |  |  | .35 |  |  |  |  |  |  |  |  |  |  |  |  |  |  |  |  |
| 42 | Uncertainty concerning the diagnosis or the therapy project of the patient |  |  |  | .42 |  |  |  |  |  |  |  |  |  |  |  |  |  |  |  |  |
| 4 | Contradictory information given by other healthcare professionals to the family |  |  |  | .42 |  |  |  |  |  |  |  | .37 |  |  |  |  |  |  |  | .39 |
| 10 | Family conflict or disagreement concerning the patient’s treatment plan |  |  |  | .45 |  |  |  |  |  |  |  | .52 |  |  |  |  |  |  |  | .53 |
| 6 | Shortage of beds in the unit |  |  |  | .56 |  |  |  |  |  |  |  | .55 |  |  |  |  |  |  |  | .6 |
| 9 | Disagreement and/or lack of coordination with other units concerning a patient’s treatment |  |  |  | .69 |  |  |  |  |  |  |  | .73 |  |  |  |  |  |  |  | .7 |
| 48 | Accumulated workloads resulting from clinical activity, training, research or teaching |  |  |  |  | .4 |  |  |  |  |  |  |  |  |  |  |  |  | .4 |  |  |
| 50 | Being on call or working nights |  |  |  |  | .49 |  |  |  |  |  | .56 |  |  |  |  |  |  | .5 |  |  |
| 36 | Continuous and heavy workload |  |  |  |  | .55 |  |  |  |  |  |  |  |  | .39 |  |  |  | .54 |  |  |
| 23 | Schedule change, overtime |  |  |  |  | .71 |  |  |  |  |  | .64 |  |  |  |  |  |  | .71 |  |  |
| 20 | Working pace or working hours hardly compatible with family or social life |  |  |  |  | .76 |  |  |  |  |  | .75 |  |  |  |  |  |  | .77 |  |  |
| 21 | Conflicts with members of the healthcare team |  |  |  |  |  | .38 |  |  |  | .46 |  |  |  |  |  |  |  |  | .43 |  |
| 17 | Negative atmosphere prevailing in the team, gossip, rumours within the team |  |  |  |  |  | .48 |  |  |  | .56 |  |  |  |  |  |  |  |  | .53 |  |
| 3 | Lack of recognition (from the patient, the family, the team, the hierarchy) |  |  |  |  |  | .51 |  |  |  | .5 |  |  |  |  |  |  |  |  | .51 |  |
| 13 | Difficulty to find my place, have my skills recognized, or voice my opinion within the team |  |  |  |  |  | .69 |  |  |  | .63 |  |  |  |  |  |  |  |  | .61 |  |
| 2 | Colleague not doing his/her work properly |  |  |  |  |  |  | .29 |  |  |  |  |  |  |  |  |  |  |  |  |  |
| 33 | Non-supportive, aggressive or delirious patient |  |  |  |  |  |  | .3 |  |  |  |  |  |  |  |  |  |  |  |  |  |
| 12 | Incomprehensible or unnecessary care relative to the patient’s situation |  |  |  |  |  |  | .34 |  |  | .34 |  |  |  |  |  |  |  |  | .4 |  |
| 11 | Too many professionals around the patient in an emergency situation |  |  |  |  |  |  | .36 |  | .34 |  |  |  |  |  |  | .32 |  |  |  |  |
| 32 | Lack of staff |  |  |  |  |  |  | .37 |  |  |  |  |  |  | .65 |  |  |  | .32 |  |  |
| 8 | Inadequate or under-equipped healthcare space or defective materials |  |  |  |  |  |  | .39 |  |  |  |  |  |  | .39 |  |  |  |  |  |  |

Note. F= Factor.

# **Table S3. Principal axis analysis with Oblimin rotation with items from the Perceived stress in intensive care units scale (PS-ICU) [**[**1**](#_ENREF_1)**,** [**2**](#_ENREF_2)**] and items specific to COVID-19 from Khalid’s scale [**[**3**](#_ENREF_3)**]**

|  |  | Dimension | | | | | |
| --- | --- | --- | --- | --- | --- | --- | --- |
|  |  | D1 (𝛼=.87) | D2 (𝛼=.80) | D3 (𝛼=.78) | D4 (𝛼=.74) | D5 (𝛼=.74) | D6 (𝛼=.66) |
| 1 | Too many professionals around the patient in an emergency situation |  |  | .381 |  |  |  |
| 2 | Having to perform tasks for which I have neither knowledge nor skills |  |  | .622 |  |  |  |
| 3 | Risk of error, fear of doing a poor job |  |  | .61 |  |  |  |
| 4 | Treating complex or serious pathologies |  |  | .746 |  |  |  |
| 5 | Having to execute care tasks quickly in emergency cases |  |  | .676 |  |  |  |
| 6 | Patient who deteriorates in an unexpected or unexplained manner |  |  | .584 |  |  |  |
| 8 | Conflicts with members of the healthcare team |  |  |  |  | .686 |  |
| 9 | Lack of recognition (from the patient. the family. the team. the hierarchy) |  |  |  |  | .434 |  |
| 10 | Negative atmosphere prevailing in the team. gossip. rumours within the team |  |  |  |  | .781 |  |
| 11 | Difficulty to find my place. have my skills recognized. or voice my opinion within the team |  |  |  |  | .45 |  |
| 12 | Being on call or working nights |  |  |  | .348 |  |  |
| 13 | Schedule changes. overtime |  |  |  | .757 |  |  |
| 14 | Working pace or working hours hardly compatible with family or social life |  |  |  | .7 |  |  |
| 15 | Continuous and heavy workload |  |  |  | .483 |  |  |
| 16 | Lack of staff |  |  |  | .355 |  |  |
| 18 | Contradictory information given by other caregivers to the family |  |  |  |  |  | .317 |
| 19 | Family conflict or disagreement concerning the patient’s treatment plan |  |  |  |  |  | .484 |
| 20 | Shortage of beds in the unit |  |  |  |  |  | .468 |
| 21 | Disagreement and/or lack of coordination with other units concerning a patient’s treatment |  |  |  |  |  | .473 |
| 22 | Patient who makes me think of someone close to me or of myself |  | .408 |  |  |  |  |
| 23 | Death of a patient with whom I had developed special ties |  | .592 |  |  |  |  |
| 24 | Having to announce a bad diagnosis to the patient or his/her family or be present when such a diagnosis is announced |  | .528 |  |  |  |  |
| 25 | Series of patient deaths in the unit over a short period |  | .513 |  |  |  |  |
| 26 | Families’ distress or emotions |  | .584 |  |  |  |  |
| 27 | Patient suffering physically or psychologically |  | .406 |  |  |  |  |
| 29k | You could transmit COVID-19 to your family or friends | .677 |  |  |  |  |  |
| 30k | Small mistake or lapse in concentration could infect you or others | .648 |  |  |  |  |  |
| 31k | Seeing patients with COVID-19 dying in front of you |  | .487 |  |  |  |  |
| 32k | Not knowing when the COVID-19 outbreak will be under control | .673 |  |  |  |  |  |
| 33k | Every time you were exposed to a new COVID-19 patient | .38 |  | **.**411 |  |  |  |
| 34k | Lack of treatment for COVID-19 | .626 |  |  |  |  |  |
| 35k | News of new cases of COVID-19 reported in TV/ newspaper | .609 |  |  |  |  |  |
| 36k | Colleagues displaying COVID-19 -like symptoms | .465 |  |  |  |  |  |
| 37k | You developed respiratory symptoms and feared that you had COVID-19 | .752 |  |  |  |  |  |
| 38k | Conflict between your duty and your own safety | .69 |  |  |  |  |  |
| 39k | You felt there were not adequate protective measures (including enough negative pressure rooms) | .466 |  |  |  |  |  |
| 40k | Recommendations and protocols evolve/change rapidly | .451 |  |  |  |  |  |
| Eigenvalue | | 4.48 | 2.52 | 3.02 | 2.13 | 1.91 | 1.63 |
| % of variance | | 12.11 | 6.80 | 8.15 | 5.75 | 5.15 | 4.41 |

*Note.* The 27 items of the PS-ICU scale were previously selected based on the strongest factorial saturation during the validation of the scale (exploratory factorial analysis using principal components and direct Oblimin rotation). The aim was to have a minimum of 4 and a maximum of 6 items per dimension.

To make the Table Seasier to read, only items with saturation >30 are presented. Items 7, 17 and 28 of the current scale were excluded from factorial analyses due to saturation <30. Item 33 was not included in the calculation of the dimensions score as it saturates doubly (D1 and D3).

# **Table S4. Items from the Perceived stress in intensive care units (PS-ICU) scale [**[**1**](#_ENREF_1)**,** [**2**](#_ENREF_2)**] and items specific to COVID-19 from Khalid’s scale [**[**3**](#_ENREF_3)**]**

| **Have you experienced this situation within your unit?** | | **Never experienced** | **I experienced this situation and...** | | | |
| --- | --- | --- | --- | --- | --- | --- |
|  |  |  | **I was not at all stressed** | **I was a little stressed** | **I was rather stressed** | **I was extremely stressed** |
| 1 (D3) | Too many professionals around the patient in an emergency situation |  |  |  |  |  |
| 2 (D3) | Having to perform tasks for which I have neither knowledge nor skills |  |  |  |  |  |
| 3 (D3) | Risk of error, fear of doing a poor job |  |  |  |  |  |
| 4 (D3) | Treating complex or serious pathologies |  |  |  |  |  |
| 5 (D3) | Having to execute care tasks quickly in emergency cases |  |  |  |  |  |
| 6 (D3) | Patient who deteriorates in an unexpected or unexplained manner |  |  |  |  |  |
| 7 | Incomprehensible or unnecessary care relative to the patient’s situation |  |  |  |  |  |
| 8 (D5) | Conflicts with members of the healthcare team |  |  |  |  |  |
| 9 (D5) | Lack of recognition (from the patient, the family, the team, the hierarchy) |  |  |  |  |  |
| 10 (D5) | Negative atmosphere prevailing in the team, gossip, rumours within the team |  |  |  |  |  |
| 11 (D5) | Difficulty to find my place, have my skills recognized, or voice my opinion within the team |  |  |  |  |  |
| 12 (D4) | Being on call or working nights |  |  |  |  |  |
| 13 (D4) | Schedule changes, overtime |  |  |  |  |  |
| 14 (D4) | Working pace or working hours hardly compatible with family or social life |  |  |  |  |  |
| 15 (D4) | Continuous and heavy workload |  |  |  |  |  |
| 16 (D4) | Lack of staff |  |  |  |  |  |
| 17 | Accumulated workloads resulting from clinical activity, training, research or teaching |  |  |  |  |  |
| 18 (D6) | Contradictory information given by other caregivers to the family |  |  |  |  |  |
| 19 (D6) | Family conflict or disagreement concerning the patient’s treatment plan |  |  |  |  |  |
| 20 (D6) | Shortage of beds in the unit |  |  |  |  |  |
| 21 (D6) | Disagreement and/or lack of coordination with other units concerning a patient’s treatment |  |  |  |  |  |
| 22 (D2) | Patient who makes me think of someone close to me or of myself |  |  |  |  |  |
| 23 (D2) | Death of a patient with whom I had developed special ties |  |  |  |  |  |
| 24 (D2) | Having to announce a bad diagnosis to the patient or his/her family or be present when such a diagnosis is announced |  |  |  |  |  |
| 25 (D2) | Series of patient deaths in the unit over a short period |  |  |  |  |  |
| 26 (D2) | Families’ distress or emotions |  |  |  |  |  |
| 27 (D2) | Patient suffering physically or psychologically |  |  |  |  |  |
| 28k | Taking care of your own colleague’s sick from COVID-19 |  |  |  |  |  |
| 29k (D1) | You could transmit COVID-19 to your family or friends |  |  |  |  |  |
| 30k (D1) | Small mistake or lapse in concentration could infect you or others |  |  |  |  |  |
| 31k (D2) | Seeing patients with COVID-19 dying in front of you |  |  |  |  |  |
| 32k (D1) | Not knowing when the COVID-19 outbreak will be under control |  |  |  |  |  |
| 33k | Every time you were exposed to a new COVID-19 patient |  |  |  |  |  |
| 34k (D1) | Lack of treatment for COVID-19 |  |  |  |  |  |
| 35k (D1) | News of new cases of COVID-19 reported in TV/ newspaper |  |  |  |  |  |
| 36k (D1) | Colleagues displaying COVID-19-like symptoms |  |  |  |  |  |
| 37k (D1) | You developed respiratory symptoms and feared that you had COVID-19 |  |  |  |  |  |
| 38k (D1) | Conflict between your duty and your own safety |  |  |  |  |  |
| 39k (D1) | You felt there were not adequate protective measures (including enough negative pressure rooms) |  |  |  |  |  |
| 40k (D1) | Recommendations and protocols evolve/change rapidly |  |  |  |  |  |

*Notes:* D1= Dimension 1- COVID-19 specific stressors; D2 = Dimension 2- Patient- and family-related emotional load; D3 = Dimension 3- Complex/risky situations and skill-related issues; D4 = Dimension 4- Workload and human-resources management issues; D5 = Dimension 5- Difficulties related to the team-working; D6 = Dimension 6- Care provided in sub-optimal or conflictual conditions. The total stress score is obtained by average the total score over the 40 items.

# **Table S5. Average scores obtained on the different scales according to epidemic intensity among students, nursing care assistants and nurses**

| Category | Students | | |  | Care assistants | | |  | Nurses | | |
| --- | --- | --- | --- | --- | --- | --- | --- | --- | --- | --- | --- |
| Epidemic Intensity | Low | High | *p* |  | Low | High | *p* |  | Low | High | *p* |
|  | Mean(SD) | Mean(SD) |  |  | Mean(SD) | Mean(SD) |  |  | Mean(SD) | Mean(SD) |  |
| GHQ12 (/12) | 4.21 (2.93) | 4.76 (3.35) | 0.404 |  | 4.11 (2.85) | 4.06 (3.16) | 0.871 |  | 3.89 (2.98) | 4.5 (3.12) | 0.001 |
| Total PS-ICU (/4) | 1.39 (0.41) | 1.46 (0.51) | 0.368 |  | 1.58 (0.57) | 1.57 (0.60) | 0.847 |  | 1.51 (0.51) | 1.62 (0.54) | <0.001 |
| Dimension 1 (/4) | 2.06 (0.64) | 2.01 (0.73) | 0.722 |  | 2.35 (0.77) | 2.26 (0.84) | 0.205 |  | 2.15 (0.71) | 2.27 (0.75) | 0.003 |
| Dimension 2 (/4) | 1.11 (0.6) | 1.37 (0.78) | 0.039 |  | 1.51 (0.91) | 1.56 (0.92) | 0.525 |  | 1.35 (0.79) | 1.52 (0.83) | <0.001 |
| Dimension 3 (/4) | 1.93 (0.84) | 1.86 (0.71) | 0.676 |  | 1.88 (0.74) | 1.84 (0.74) | 0.567 |  | 1.92 (0.74) | 1.96 (0.73) | 0.31 |
| Dimension 4 (/4) | 1.22 (0.7) | 1.3 (0.78) | 0.579 |  | 1.46 (0.76) | 1.44 (0.72) | 0.734 |  | 1.46 (0.69) | 1.63 (0.77) | <0.001 |
| Dimension 5 (/4) | 0.88 (0.9) | 0.95 (0.92) | 0.699 |  | 0.96 (0.94) | 0.94 (0.89) | 0.823 |  | 0.96 (0.85) | 1 (0.9) | 0.413 |
| Dimension 6 (/4) | 0.33 (0.5) | 0.69 (0.66) | 0.001 |  | 0.63 (0.73) | 0.75 (0.75) | 0.061 |  | 0.66 (0.72) | 0.81 (0.75) | <0.001 |
| Coping strategies |  |  |  |  |  |  |  |  |  |  |  |
| Social support (/4) | 2.86 (0.89) | 2.45 (0.8) | 0.015 |  | 2.67 (0.81) | 2.57 (0.86) | 0.193 |  | 2.6 (0.81) | 2.68 (0.84) | 0.076 |
| Problem solving (/4) | 2.72 (0.85) | 2.41 (0.97) | 0.076 |  | 2.7 (0.93) | 2.65 (0.92) | 0.599 |  | 2.57 (0.91) | 2.69 (0.92) | 0.018 |
| Avoidance (/4) | 2.54 (0.87) | 2.53 (0.97) | 0.932 |  | 2.49 (1) | 2.64 (1.03) | 0.09 |  | 2.59 (0.95) | 2.7 (0.96) | 0.037 |
| Positive thinking (/4) | 2.62 (1.06) | 2.72 (1.01) | 0.634 |  | 2.86 (0.92) | 2.8 (0.95) | 0.543 |  | 2.89 (0.9) | 2.8 (0.91) | 0.092 |

*Note.* Figures represent averages with standard deviations in parentheses. Dimension 1: COVID-19 specific stressors; Dimension 2: Patient- and family-related emotional load; Dimension 3: Complex/risky situations and skill-related issues; Dimension 4: Workload and human-resources management issues; Dimension 5: Difficulties related to the team-working; Dimension 6: Care provided in sub-optimal or conflictual conditions; PS-ICU: Perceived stress scale in intensive care unit; GHQ-12: 12-item General Health Questionnaire.

# **Table S6. Average scores obtained on the different scales according to epidemic intensity among Nursing managers, residents and physicians**

| Category | Nursing managers | | |  | Residents | | |  | Physicians | | |
| --- | --- | --- | --- | --- | --- | --- | --- | --- | --- | --- | --- |
| Epidemic intensity | Low | High | *p* |  | Low | High | *p* |  | Low | High | *p* |
|  | Mean(SD) | Mean(SD) |  |  | Mean(SD) | Mean(SD) |  |  | Mean(SD) | Mean(SD) |  |
| GHQ12 (/12) | 4.12 (2.01) | 3.72 (2.36) | 0.483 |  | 3.27 (2.53) | 4.21 (3.03) | 0.046 |  | 3.27 (2.45) | 4 (2.9) | 0.032 |
| Total PS-ICU (/4) | 1.32 (0.61) | 1.43 (0.47) | 0.438 |  | 1.35 (0.39) | 1.55 (0.52) | 0.007 |  | 1.29 (0.47) | 1.45 (0.46) | 0.005 |
| Dimension 1 (/4) | 1.98 (0.86) | 2.05 (0.63) | 0.73 |  | 1.49 (0.54) | 1.7 (0.56) | 0.027 |  | 1.62 (0.68) | 1.81 (0.64) | 0.023 |
| Dimension 2 (/4) | 0.91 (0.82) | 1.06 (0.75) | 0.471 |  | 1.47 (0.73) | 1.74 (0.78) | 0.034 |  | 1.19 (0.62) | 1.43 (0.67) | 0.003 |
| Dimension 3 (/4) | 0.95 (0.66) | 1.31 (0.78) | .049 |  | 1.77 (0.7) | 1.92 (0.64) | 0.174 |  | 1.51 (0.7) | 1.56 (0.64) | 0.637 |
| Dimension 4 (/4) | 1.93 (0.88) | 1.97 (0.81) | 0.839 |  | 1.36 (0.73) | 1.64 (0.82) | 0.030 |  | 1.46 (0.77) | 1.56 (0.72) | 0.278 |
| Dimension 5 (/4) | 1.44 (0.91) | 1.27 (0.91) | 0.444 |  | 0.87 (0.81) | 0.96 (0.91) | 0.509 |  | 0.81 (0.82) | 1.01 (0.94) | 0.059 |
| Dimension 6 (/4) | 0.76 (0.79) | 0.76 (0.63) | 0.988 |  | 0.91 (0.7) | 1.07 (0.74) | 0.179 |  | 0.87 (0.78) | 1.1 (0.76) | 0.019 |
| Coping strategies |  |  |  |  |  |  |  |  |  |  |  |
| Social support (/4) | 2.6 (0.76) | 2.87 (0.92) | 0.206 |  | 2.39 (0.83) | 2.61 (0.78) | 0.118 |  | 2.14 (0.79) | 2.41 (0.79) | 0.009 |
| Problem solving (/4) | 3.12 (0.91) | 3.03 (0.9) | 0.697 |  | 2.52 (0.93) | 2.68 (0.83) | 0.304 |  | 2.73 (0.91) | 2.84 (0.9) | 0.317 |
| Avoidance (/4) | 2.69 (0.88) | 3.1 (0.91) | 0.076 |  | 2.37 (0.96) | 2.72 (0.9) | 0.030 |  | 2.31 (1.03) | 2.51 (0.9) | 0.107 |
| Positive thinking (/4) | 3.15 (0.73) | 3.26 (0.79) | 0.593 |  | 2.75 (0.94) | 2.81 (0.84) | 0.669 |  | 2.75 (0.83) | 2.85 (0.86) | 0.378 |

*Note.* Figures represent averages with standard deviations in parentheses. Dimension 1: COVID-19 specific stressors; Dimension 2: Patient- and family-related emotional load; Dimension 3: Complex/risky situations and skill-related issues; Dimension 4: Workload and human-resources management issues; Dimension 5: Difficulties related to the team-working; Dimension 6: Care provided in sub-optimal or conflictual conditions; PS-ICU: Perceived stress scale in intensive care unit; GHQ-12: 12-item General Health Questionnaire.

# **Table S7. Number of participants with a mental health disorder (GHQ-12 ≥ 3) according to occupational category and epidemic intensity**

|  |  |  | Epidemic intensity | | |  |
| --- | --- | --- | --- | --- | --- | --- |
|  | All (n=1468) |  | Low (n=503) |  | High (n=965) | |
| Students | 70 |  | 19 |  | 51 |  |
| Nursing care assistants | 269 |  | 120 |  | 149 |  |
| Nurses | 810 |  | 279 |  | 531 |  |
| Nursing managers | 45 |  | 21 |  | 24 |  |
| Residents | 88 |  | 23 |  | 65 |  |
| Physicians | 186 |  | 41 |  | 145 |  |

# **Table S8. Average scores on the different scales among professionals with a mental health disorder (GHQ-12 ≥ 3)**

|  | Total | | | Epidemic intensity | | | | Welch’s F | | p | |  |
| --- | --- | --- | --- | --- | --- | --- | --- | --- | --- | --- | --- | --- |
|  |  | | | Low | | High | |  | |  | |  |
| Mental health (/12) | | 5.90 (2.38) | | | 5.61 (2.28) | | 6.05 (2.42) | | 11.33 | | 0.001 | |
| Overall perceived stress (/4) | 1.72 (0.53) | | 1.63 (0.53) | | | 1.77 (0.52) | | 24.30 | | <0.001 | |  |
| Dimension 1 (/4) | 2.34 (0.74) | | 2.30 (0.74) | | | 2.36 (0.74) | | 2.26 | | 0.133 | |  |
| Dimension 2 (/4) | 1.62 (0.85) | | 1.46 (0.86) | | | 1.72 (0.83) | | 28.63 | | <0.001 | |  |
| Dimension 3 (/4) | 2.01 (0.76) | | 1.95 (0.77) | | | 2.04 (0.75) | | 3.84 | | 0.050 | |  |
| Dimension 4 (/4) | 1.76 (0.78) | | 1.65 (0.77) | | | 1.82 (0.79) | | 16.18 | | <0.001 | |  |
| Dimension 5 (/4) | 1.17 (0.95) | | 1.09 (0.93) | | | 1.21 (0.96) | | 4.91 | | 0.027 | |  |
| Dimension 6 (/4) | 0.91 (0.80) | | 0.75 (0.79) | | | 0.99 (0.79) | | 30.84 | | <0.001 | |  |
| Coping strategies |  | |  | | |  | |  | |  | |  |
| Social support (/4) | 2.79 (0.77) | | 2.75 (0.75) | | | 2.81 (0.78) | | 2.51 | | 0.114 | |  |
| Problem solving (/4) | 2.81 (0.86) | | 2.77 (0.85) | | | 2.82 (0.87) | | 1.03 | | 0.311 | |  |
| Avoidance (/4) | 2.80 (0.90) | | 2.73 (0.90) | | | 2.84 (0.90) | | 4.92 | | 0.027 | |  |
| Positive-thinking (/4) | 2.77 (0.89) | | 2.81 (0.87) | | | 2.75 (0.90) | | 1.26 | | 0.261 | |  |

*Note.* Mean and standard deviation are reported. Dimension 1: COVID-19 specific stressors; Dimension 2: Patient- and family-related emotional load; Dimension 3: Complex/risky situations and skill-related issues; Dimension 4: Workload and human-resources management issues; Dimension 5: Difficulties related to the team-working; Dimension 6: Care provided in sub-optimal or conflictual conditions; PSI-CU: Perceived stress scale in intensive care unit; GHQ-12: 12-item General Health Questionnaire.

# **Table S9. Correlations between questionnaire scores.**

|  | GHQ-12 | Total PS-ICU | D1 | D2 | D3 | D4 | D5 | D6 | Social support | Problem solving | Avoidance |
| --- | --- | --- | --- | --- | --- | --- | --- | --- | --- | --- | --- |
| Total PS-ICU | 0.52^***^ | 1 |  |  |  |  |  |  |  |  |  |
| Dimension 1 (/4) | 0.45^***^ | 0.76^***^ | 1 |  |  |  |  |  |  |  |  |
| Dimension 2 (/4) | 0.32^***^ | 0.75^***^ | 0.45^***^ | 1 |  |  |  |  |  |  |  |
| Dimension 3 (/4) | 0.40^***^ | 0.66^***^ | 0.37^***^ | 0.42^***^ | 1 |  |  |  |  |  |  |
| Dimension 4 (/4) | 0.44^***^ | 0.72^***^ | 0.45^***^ | 0.42^***^ | 0.38^***^ | 1 |  |  |  |  |  |
| Dimension 5 (/4) | 0.39^***^ | 0.59^***^ | 0.27^***^ | 0.30^***^ | 0.33^***^ | 0.44^***^ | 1 |  |  |  |  |
| Dimension 6 (/4) | 0.20^***^ | 0.59^***^ | 0.24^***^ | 0.45^***^ | 0.26^***^ | 0.41^***^ | 0.38^***^ | 1 |  |  |  |
| Social support | 0.36^***^ | 0.39^***^ | 0.38^***^ | 0.30^***^ | 0.25^***^ | 0.26^***^ | 0.18^***^ | 0.11^***^ | 1 |  |  |
| Problem solving | 0.19^***^ | 0.25^***^ | 0.25^***^ | 0.18^***^ | 0.12^***^ | 0.17^***^ | 0.11^***^ | 0.09^***^ | 0.34^***^ | 1 |  |
| Avoidance | 0.30^***^ | 0.33^***^ | 0.30^***^ | 0.24^***^ | 0.21^***^ | 0.24^***^ | 0.19^***^ | 0.12^***^ | 0.35^***^ | 0.36^***^ | 1 |
| Positive thinking | -0.16^***^ | -0.06^**^ | -0.07^***^ | -0.02 | -0.06^**^ | -0.05^*^ | -0.03 | <0.001 | 0.07^***^ | 0.18^***^ | 0.20^***^ |

*Note*. D1 = Dimension 1- COVID-19 specific stressors; D2 = Dimension 2- Patient- and family-related emotional load; D3 = Dimension 3- Complex/risky situations and skill-related issues; D4 = Dimension 4- Workload and human-resources management issues; D5 = Dimension 5- Difficulties related to the team-working; D6 = Dimension 6- Care provided in sub-optimal or conflictual condition; PS-ICU: Perceived stress scale in intensive care unit; GHQ-12: 12-item General Health Questionnaire.

^***^p<.001; ^**^p<.01; ^*^ p<.05

**Additional file 1: Figure S1. Graphical representation of the average scores on the different dimensions of the PS-ICU among all professionals.**

Note. Mean and standard error of the mean are reported.

F (4.65, 12292.35) = 1728.54, p<.001. Bonferroni post-hoc comparisons showed that mean dimension scores are significantly different two by two, *p*<0.001.


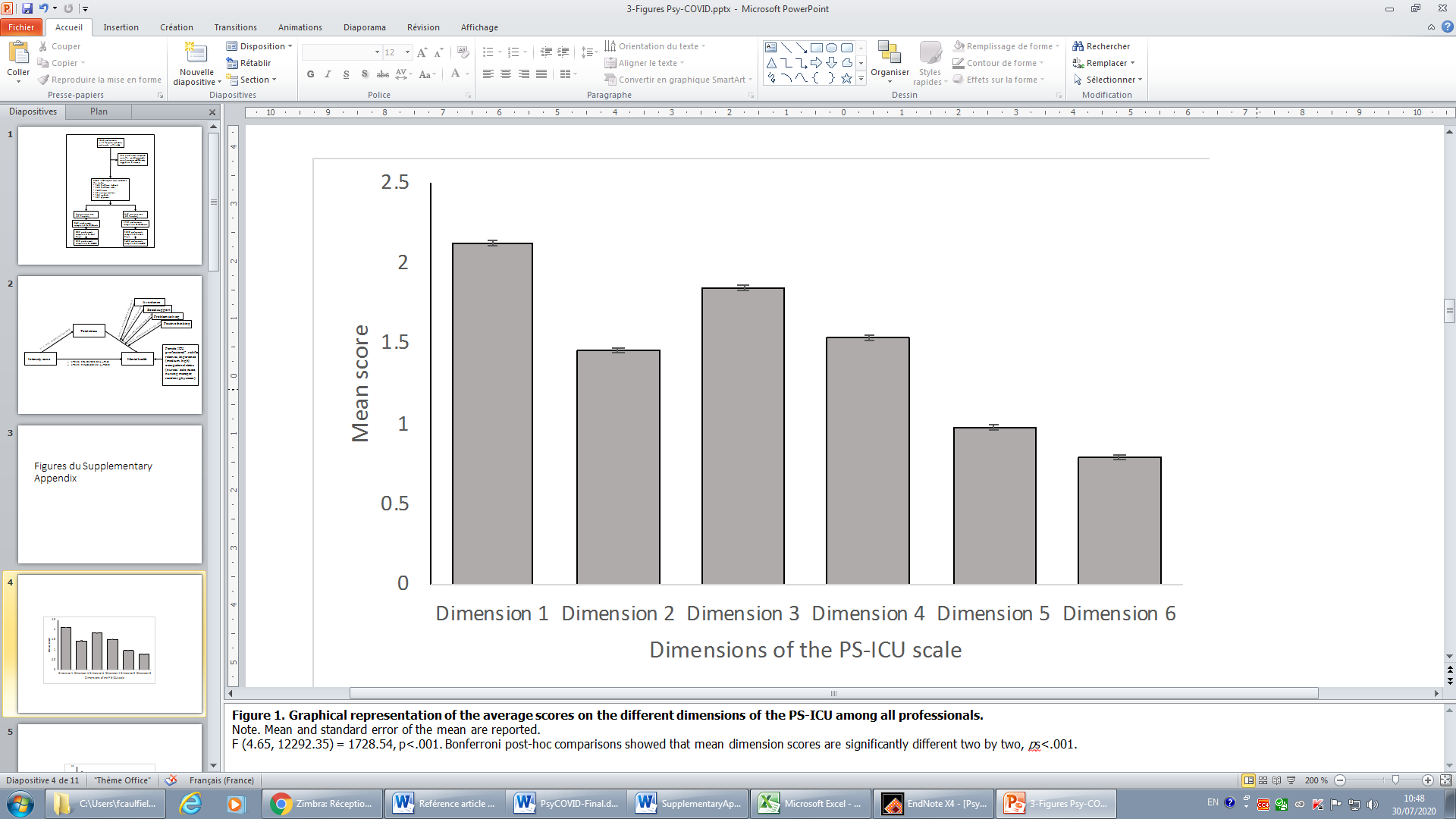


**Additional file 1: Figure S2. Graphical representation of the average scores for the different dimensions of the PS-ICU among all professionals according to the epidemic zone.**

Note. Mean and standard error of the mean are reported.

Interaction between epidemic intensity zone and stress factors F (4.65, 12288.41) = 6.77, p<0.001.

Pairwise comparisons (Bonferroni) indicate that average scores on dimensions D2, D4 and D6 differ significantly between high and low-intensity zones (*p*<0.001).

Within each zone, all factors differ significantly in 2 by 2 pairwise comparison, p<0.05 for each.


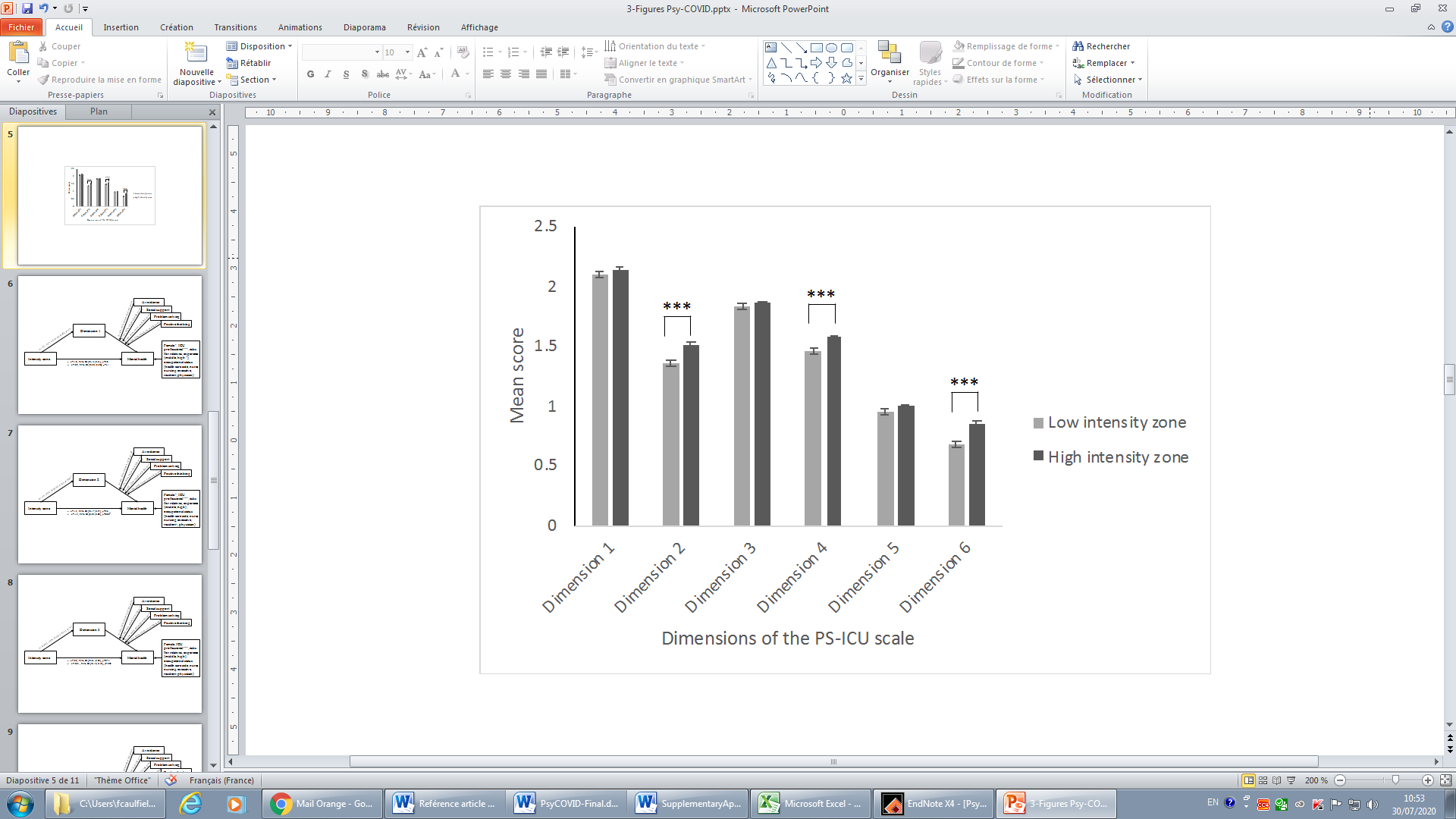


**Additional file 1: Figure S3. Mediation and moderation analysis of the relationship between the epidemic intensity zone and mental health through Dimension 1 scores (*COVID-19 specific items*).**

Female: b= 0.29, 95%-CI=0.03, 0.55, p=0.029; ICU professional: b= 0.56, 95%-CI= 0.3, 0.82, p<0.001; High experience: b= -0.30, 95%-CI= -0.58, -0.02, p=0.036


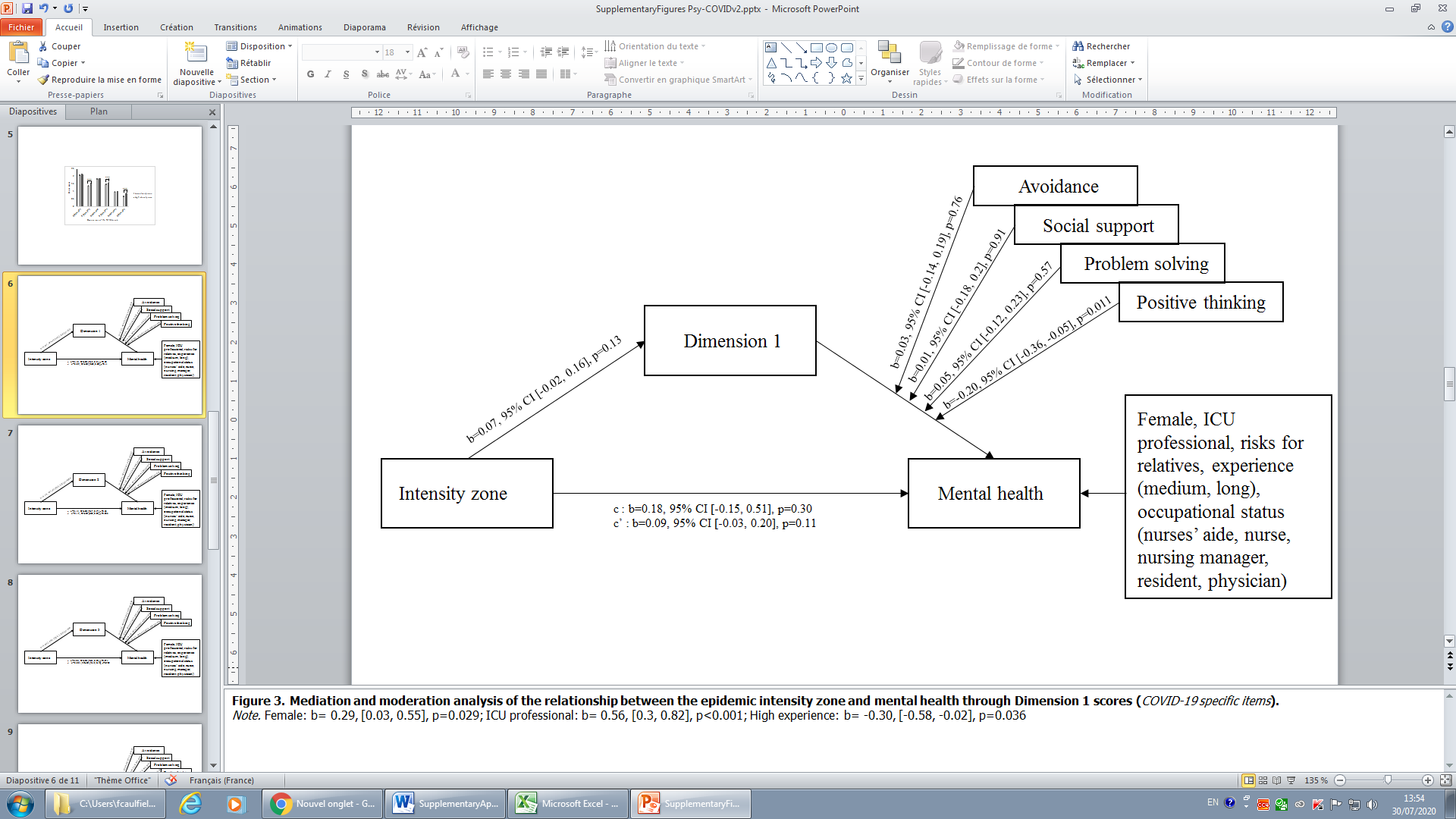


**Additional file 1: Figure S4.** **Mediation and moderation analysis of the relationship between the epidemic intensity zone and mental health through Dimension 2 scores *(Patient- and family-related emotional load)***

Female: b= 0.29, 95%-CI= 0.02, 0.55, p=0.033; ICU professional: b= 0.83, 95%-CI= 0.56, 1.1, p<0.001


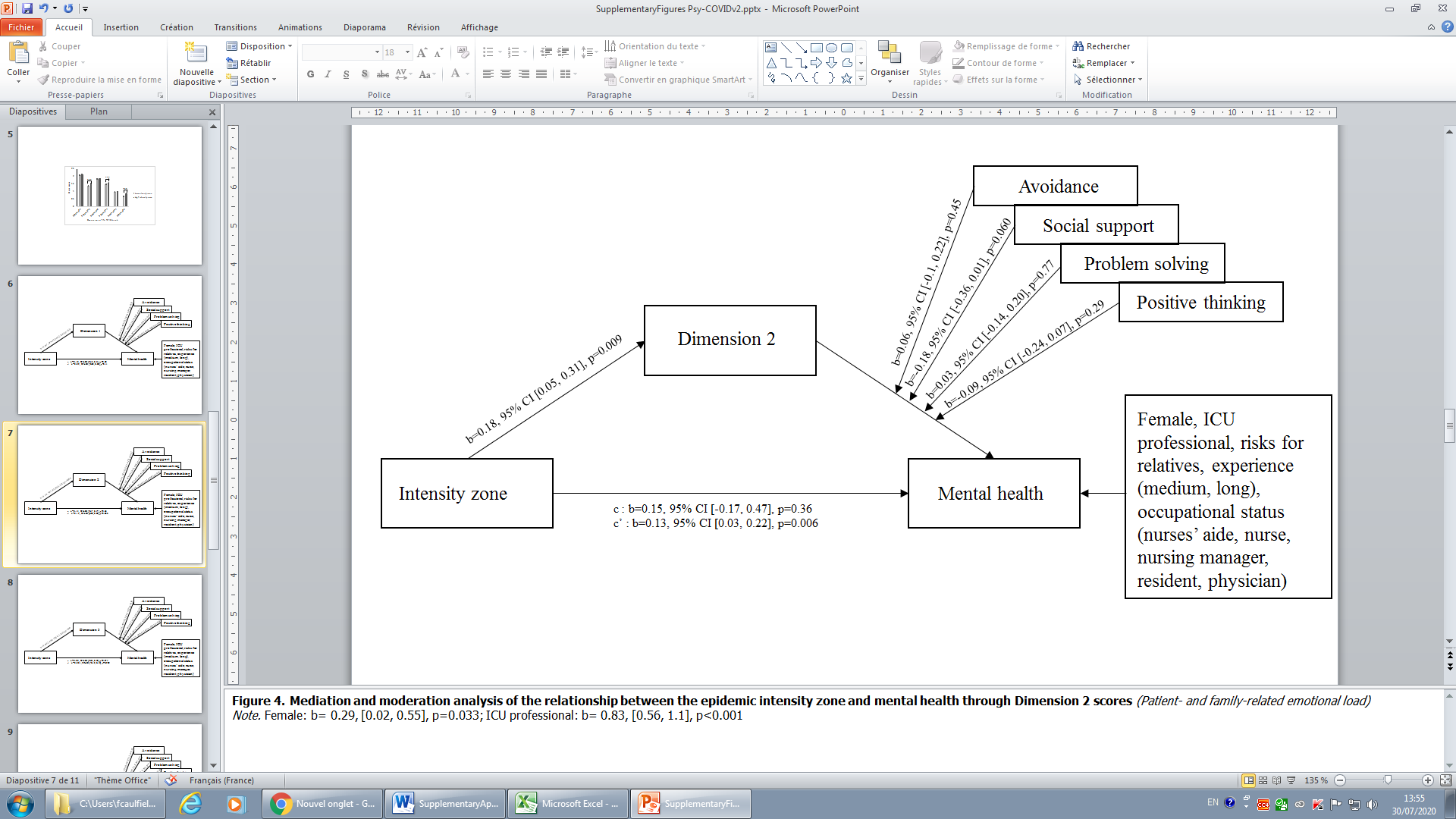


**Additional file 1: Figure S5. Mediation and moderation analysis of the relationship between the epidemic intensity zone and mental health through dimension 3 scores (*Complex/risky situations and skill-related issues*).**

ICU professional: b= 0.47, 95%-CI= 0.21, 0.72, p<0.001


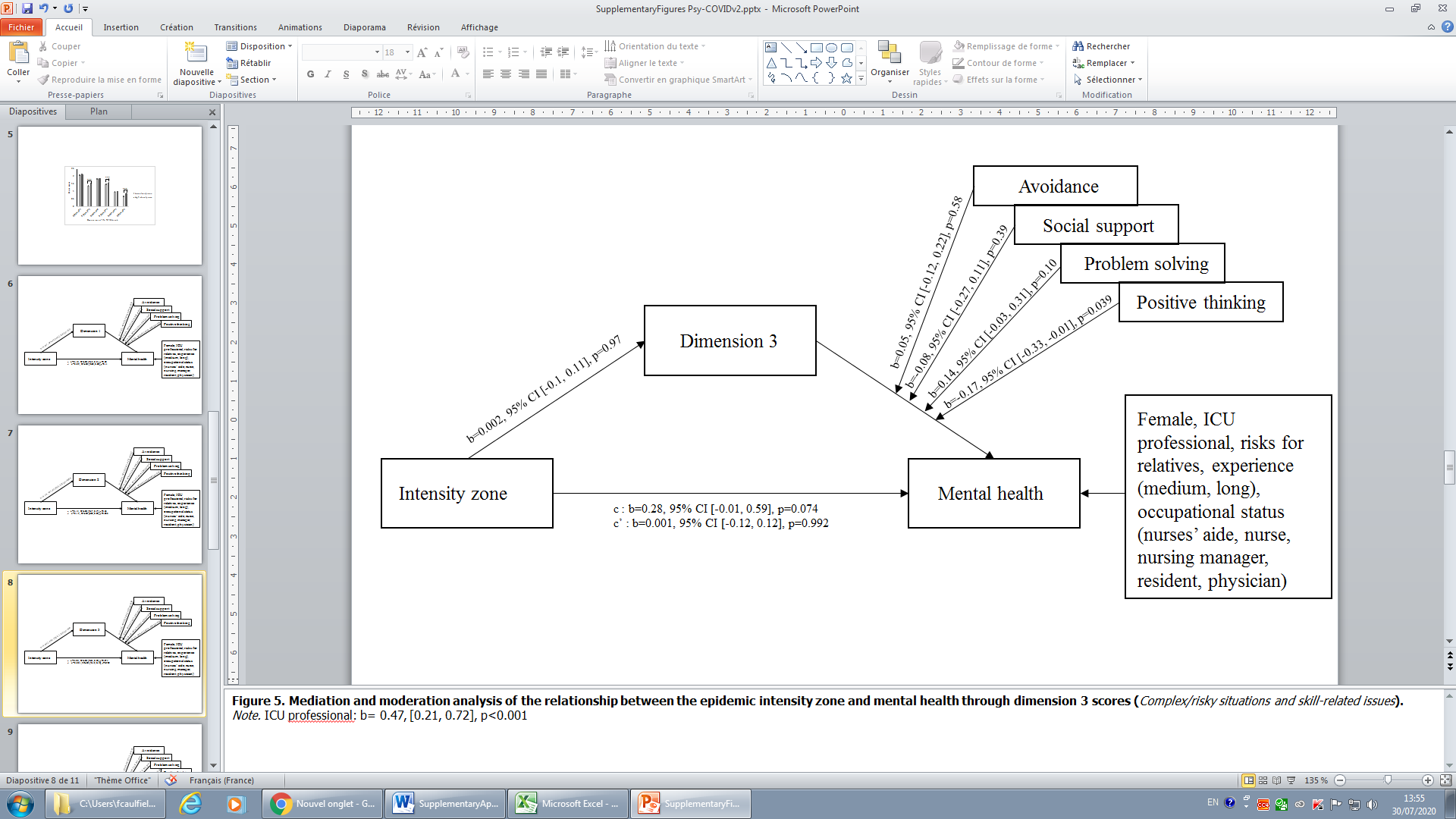


**Additional file 1: Figure S6. Mediation and moderation analysis of the relationship between the epidemic intensity zone and mental health through Dimension 4 scores (*Workload and human-resources management issues)*.**

Female: b= 0.33, 95%-CI= 0.08, 0.59, p=0.010; ICU professional: b= 0.72, 95%-CI= 0.47, 0.98, p<0.001; Medium experience: b= -0.32, 95%-CI= -0.6, -0.03, p=0.027; Nursing manager: b= -1.5, 95%-CI= -2.36, -0.64, p<0.001; resident: b= -0.77, 95%-CI= -1.46, -0.09, p=0.028.


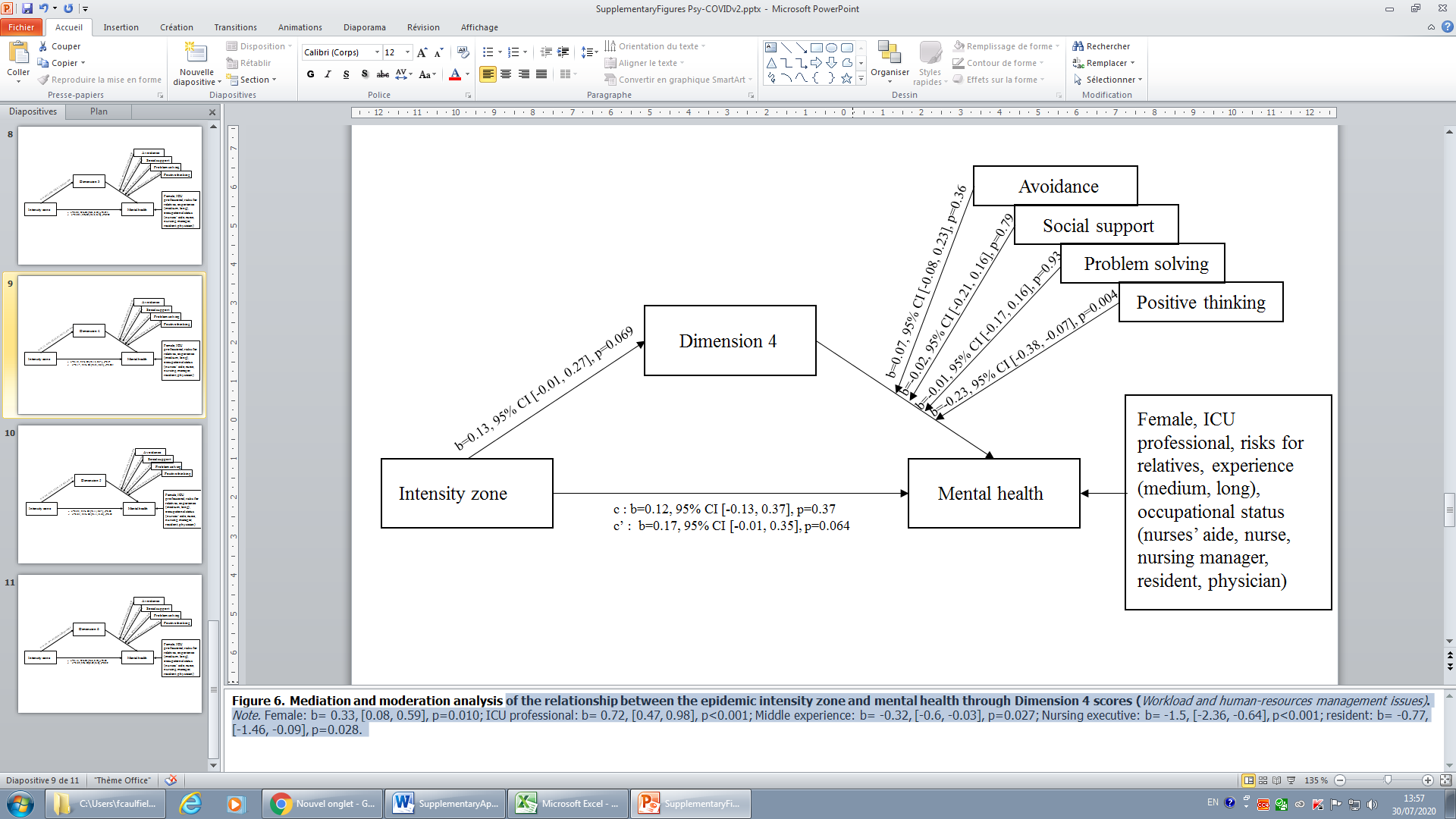


**Additional file 1: Figure S7. Mediation and moderation analysis of the relationship between the epidemic intensity zone and mental health through Dimension 5 scores (*Difficulties related to the team-working*).**

Female: b= 0.38, 95%-CI= 0.12, 0.64, p=0.003; ICU professional: b= 0.79, 95%-CI= 0.53, 1.04, p<0.001


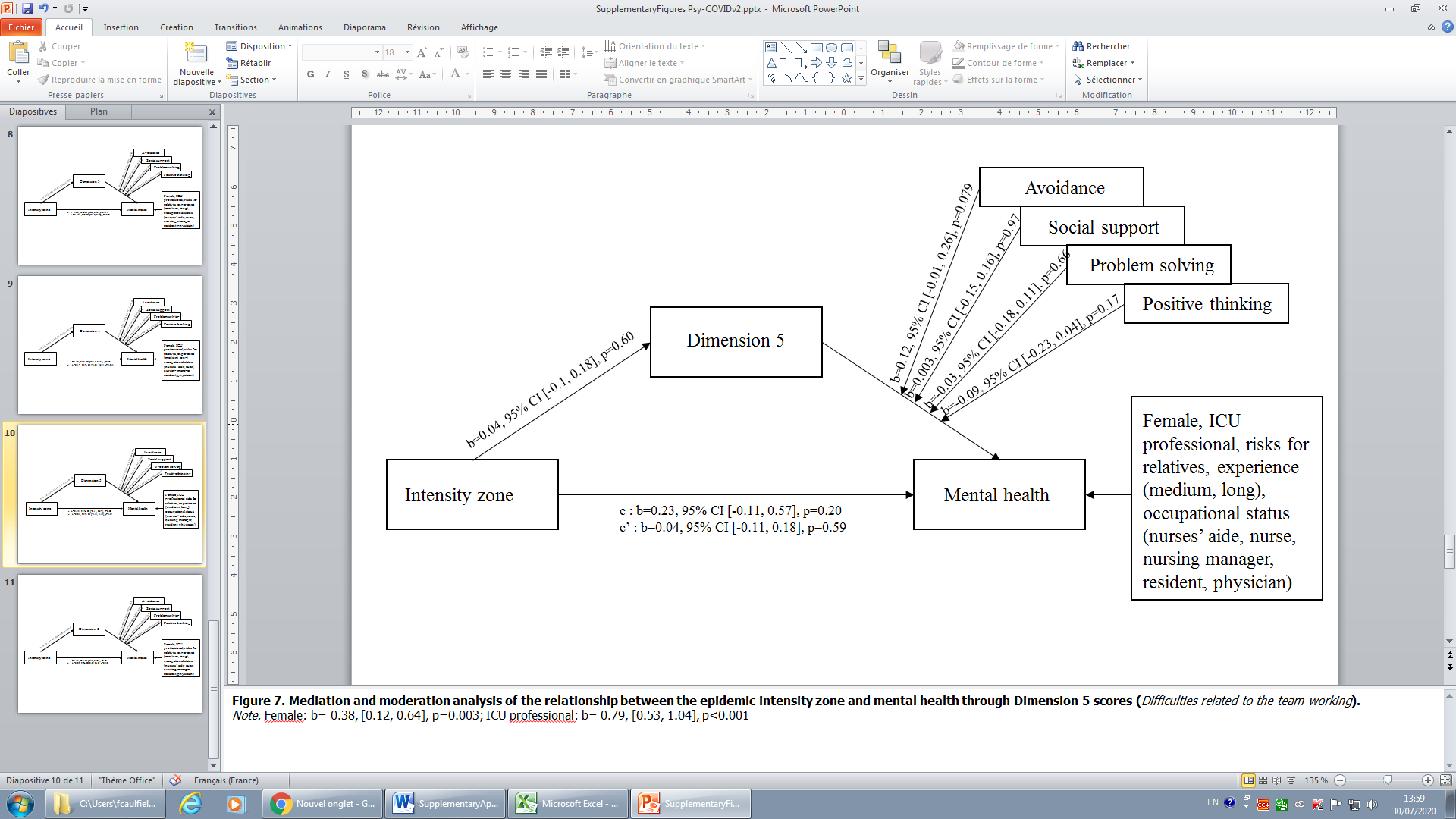


**Additional file 1: Figure S8. Mediation and moderation analysis of the relationship between the epidemic intensity zone and mental health through Dimension 6 scores (*Care provided in sub-optimal or conflictual conditions*).**

Female: b= 0.48, 95%-CI= 0.21, 0.74, p<0.001; ICU professional: b= 0.78, 95%-CI= 0.51, 1.05, p<0.001


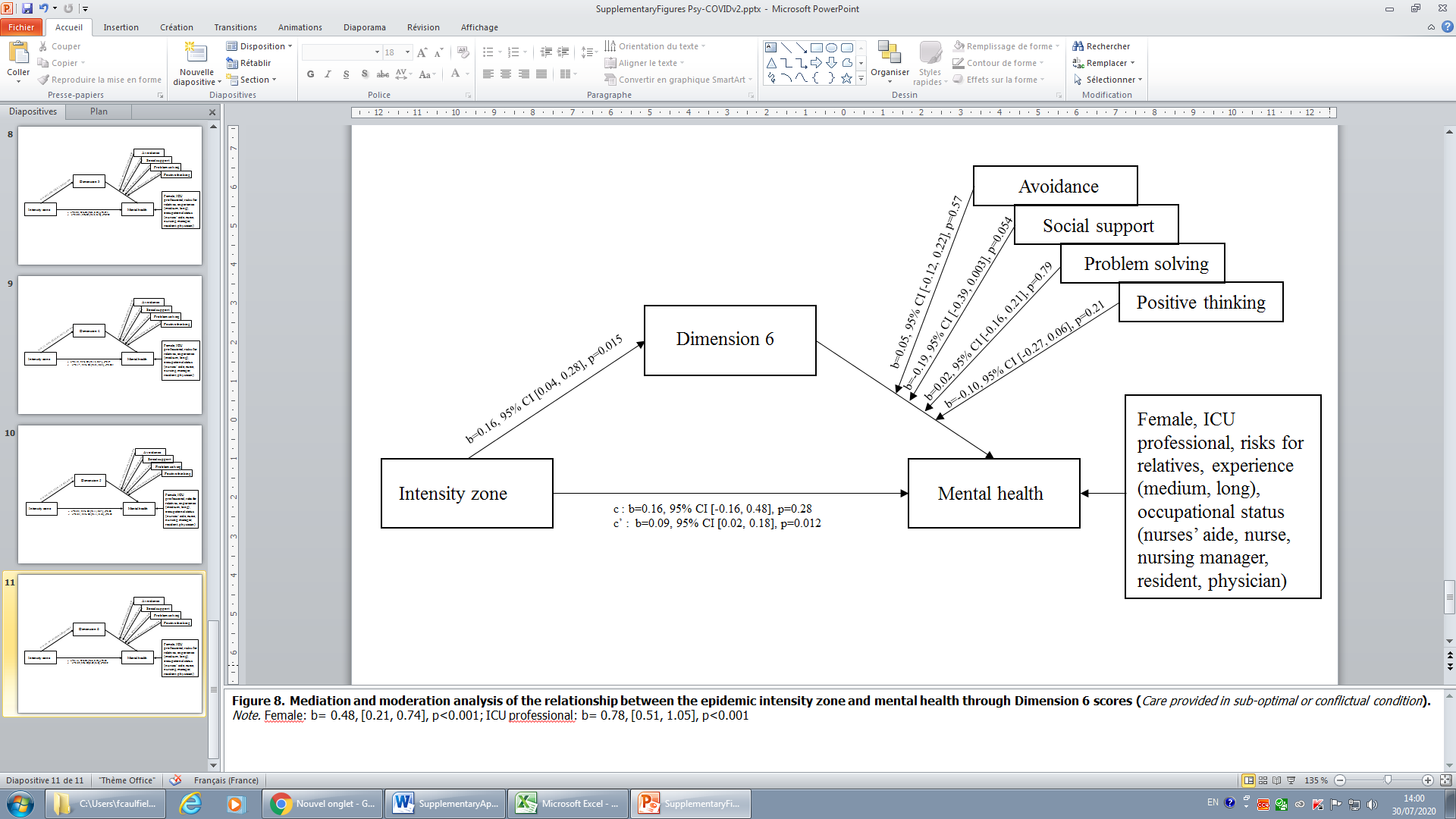


# **Additional file 1: Appendix References**

1. Fournier A, Lheureux F, Genet M, Martin Delgado MC, Bocci MG, Prestifilippo A, et al. Construction of a stress scale specific to intensive care units: the PS-ICU scale. Proceedings of Reanimation 2020, the French Intensive Care Society International Congress. Ann Intensive Care. 2020;10:16.

2. Laurent A, Lheureux F, Genet M, Martin Delgado MC, Bocci MG, Prestifilippo A, et al. Scales Used to Measure Job Stressors in Intensive Care Units: Are They Relevant and Reliable? A Systematic Review. Front Psychol. 2020;11:245.

3. Khalid I, Khalid TJ, Qabajah MR, Barnard AG, Qushmaq IA. Healthcare Workers Emotions, Perceived Stressors and Coping Strategies During a MERS-CoV Outbreak. Clin Med Res. 2016;14:7-14.
